# Analysis of a mechanistic Markov model for gene duplicates evolving under subfunctionalization

**DOI:** 10.1186/s12862-016-0848-0

**Published:** 2017-01-31

**Authors:** Tristan L. Stark, David A. Liberles, Barbara R. Holland, Małgorzata M. O’Reilly

**Affiliations:** 1 School of Physical Sciences, University of Tasmania, Churchill Ave, Hobart, 7001 Australia; 2 Center for Computational Genetics and Genomics and Department of Biology, Temple University, Philadelphia, 19122 USA

**Keywords:** Continuous-time Markov chain (CTMC), Phase-type distribution, Subfunctionalization, Nonfunctionalization, Pseudogenization, Neofunctionalization, Gene duplication

## Abstract

**Background:**

Gene duplication has been identified as a key process driving functional change in many genomes. Several biological models exist for the evolution of a pair of duplicates after a duplication event, and it is believed that gene duplicates can evolve in different ways, according to one process, or a mix of processes. Subfunctionalization is one such process, under which the two duplicates can be preserved by dividing up the function of the original gene between them. Analysis of genomic data using subfunctionalization and related processes has thus far been relatively coarse-grained, with mathematical treatments usually focusing on the phenomenological features of gene duplicate evolution.

**Results:**

Here, we develop and analyze a mathematical model using the mechanics of subfunctionalization and the assumption of Poisson rates of mutation. By making use of the results from the literature on the Phase-Type distribution, we are able to derive exact analytical results for the model.

The main advantage of the mechanistic model is that it leads to testable predictions of the phenomenological behavior (instead of building this behavior into the model a priori), and allows for the estimation of biologically meaningful parameters. We fit the survival function implied by this model to real genome data (*Homo sapiens*, *Mus musculus*, *Rattus norvegicus* and *Canis familiaris*), and compare the fit against commonly used phenomenological survival functions. We estimate the number of regulatory regions, and rates of mutation (relative to silent site mutation) in the coding and regulatory regions.

We find that for the four genomes tested the subfunctionalization model predicts that duplicates most-likely have just a few regulatory regions, and the rate of mutation in the coding region is around 5-10 times greater than the rate in the regulatory regions. This is the first model-based estimate of the number of regulatory regions in duplicates.

**Conclusions:**

Strong agreement between empirical results and the predictions of our model suggest that subfunctionalization provides a consistent explanation for the evolution of many gene duplicates.

**Electronic supplementary material:**

The online version of this article (doi:10.1186/s12862-016-0848-0) contains supplementary material, which is available to authorized users.

## Background

In this paper, we consider the evolution of a pair of gene duplicates following a duplication event which gives rise to two perfect copies of the original gene. Gene duplication was first presented as an important process by Ohno [1], who postulated that the emergence of new functions in genomes was enabled by gene duplication. Ohno [1] claimed that duplication relaxed selective pressures on proteins and enabled mutations to accumulate, leading to the eventual process of neofunctionalization. Gene duplication has since been identified as a common occurrence in sequenced genomes [2], and as an important contributor to genome diversification [3, 4].

*Subfunctionalization* was first analyzed in a series of papers by Force and Lynch [5–7]. It is a process of subdividing functions from the ancestral state between the duplicated gene copies, which allows for both copies of the gene to be preserved by selective pressure without the need to invoke positive selection (as in the neofunctionalization process). To model the evolution of gene duplicates, subfunctionalization is taken together with *pseudogenization*, a process in which genes lose all functionality, and are effectively lost to the genome. These competing processes describe the ultimate fate of duplicates under subfunctionalization; the copies will either subfunctionalize, in which case both are preserved by selective pressure, or one of the copies will eventually pseudogenize, and the other copy will be preserved.

Hughes and Liberles [8] sought to build upon the work in Force and Lynch [6] and Lynch and Conery [2, 9] to generate models for neofunctionalization and subfunctionalization. In these works [2, 6, 8, 9], the expected behaviour of duplicates evolving under sub/neofunctionalization is characterized by inspection of the mechanisms of both processes. Survival/hazard functions were chosen for their conformity to the predictions implied by the characterization and used for subsequent analysis. However, the parameters in these survival/hazard functions do not uniquely characterize processes in the mechanistic framework. Here we perform a complete mechanistic analysis of subfunctionalization. The mechanistic approach has two main advantages in comparison to fitting phenomenological functions (a) it leads to testable predictions about the shape of the survival function under the subfunctionalization model (b) it allows estimation of biologically meaningful parameters such as null mutation rates and the number of functions that can be partitioned. The main contributions of this work are:

- Development of a continuous-time Markov chain model for the subfunctionalization process.
- Analysis of the shape properties, long and short-term behaviour of pseudogenization rate, and interpretation of what this means in terms of biological predictions of the subfunctionalization process.
- Fitting the model to genome data for four mammalian species.
- The first subfunctionalization-model-based estimates of the relative rate of fixation of null mutations both in the coding and regulatory regions of gene duplicates.
- The first model-based estimates of the likely numbers of regulatory regions to exist in gene duplicates.

In the section “A continuous-time Markov chain model for Subfunctionalization” we contstruct the model, justify the various transition rates by analyzing the mechanics of the subfunctionalization process, and note some important structural features of the model.

In the section “The pseudogenization cause-specific hazard rate” we introduce the pseudogenization (and subfunctionalization) cause-specific hazard rates. This gives the instantaneous rate of pseudogenization (or subfunctionalization) under the assumption that neither subfunctionalization, nor pseudogenization has occurred so-far. We offer a minor correction to the approximation applied by Hughes and Liberles [8].

In the section “Pseudogenization rates” we define and derive results for what we call the *pseudogenization rate*. This gives the instantaneous rate of pseudogenization *without* the assumption that subfunctionalization has not yet occurred (as in the standard hazard rate analysis). We argue that this rate is more useful in the context of subfunctionalization than the pseudogenization cause-specific hazard rate. We analyze the shape properties of this function, showing that subfunctionalization predicts qualitative behaviour which is consistent with the empirically observed curve [8].

In the section “Subfunctionalization survival function and Poisson duplication” we introduce a simple model for the underlying duplication process. We apply this, together with the survival function implied by our subfunctionalization model, to derive an equation for the likelihood of observing some count of duplicates at a particular time. By taking the product of these likelihoods over each of the time points represented in a data set, it is possible to estimate parameters for both the subfunctionalization model, and the duplication model.

In the section “Shape properties of the pseudogenization rate functions” we examine the shape properties of the pseudogenization rate functions under some different parameterizations. We find that the rate function is sigmoidally shaped, but under certain parameterizations can appear exponential-like in shape.

In the section “Fitting the model to genome data” we fit the model to the genomic data set analyzed in Hughes and Liberles [8], and derive some relevant results. The mechanistic nature of our model means that this fitting provides direct estimates of the rates of mutation in the genome relative to synonymous site mutation, as well as the number of regulatory regions that duplicate genes are likely to have.

In the “Discussion” we consider some biological implications of this analysis. By analyzing the shape properties of the pseudogenization rate function, we rethink the predictions on gene duplicate survival implied by the subfunctionalization process. While our analysis agrees in part with previous characterizations, particularly due to Hughes and Liberles [8], we highlight several points of difference. Most notably, we discuss the finding that our model does not necessarily lead to the broadly concave decline in pseudogenization rate thought to characterize subfunctionalization [8, 10–12]. We see that, under certain parameter sets, the model can lead to rapid convex decline in the hazard rate which would be associated with rapid subfunctionalization and a low risk of nonfunctionalization; and we suggest some possible candidate genes which might exhibit this behaviour.

In the “Conclusions” we discuss the results of the mathematical and empirical analysis, and what this means for the biological process of subfunctionalization. We summarize our goals for modeling gene duplication with reference to the development of an overall mathematical model for gene duplication, incorporating all of the major biological processes.

Much of the mathematical detail is omitted from the main text, and is included in Additional file 1: Section A of Additional file 1: makes use of the embedded discrete-time Markov chain to derive various probabilities of interest, including the probability of absorption by the time of the *n* ^th^ mutation.

In Section B of Additional file 1 we consider the cause-specific hazard rates, and derive various measures of interest including the probability of absorption before time *t*, mean time to absorption and the *k* ^th^ moments of time to absorption.

Section C of Additional file 1 contains the derivation of the main rate of interest in this work, the pseudogenization rate, as well as the average pseudogenization rate where the number of regulatory regions is treated as a random variable.

In Additional file 1: Section E we compare the qualitative features of the model detailed in this work to two existing phenomenological approximations. We also outline a method for retro-fitting our model to the model of Teufel et al. [11].

Sections F and G of Additional file 1: contain proofs of two important results which are used in the main body of the text. We consider the general case of an absorbing Continuous-time Markov chain (CTMC) with finite state space, and derive limits of the cause-specific hazard rate (Additional file 1: Section F), and an analogue of the pseudogenization rate discussed throughout the main body of this work (Additional file 1: Section G).

## Methods

### A continuous-time Markov chain model for Subfunctionalization

In this section, we define the model which is central to this paper. The model is a continuous-time Markov chain (CTMC), with a structure which is very similar to the phase-type distribution, [13] which has been widely studied in the probability modeling literature, but has so-far seen little or no use in evolutionary biology. In later sections, we will exploit this structural similarity in the derivation of hazard rates and other measures. In Additional file 1: Section A, we also perform an analysis of the embedded discrete-time Markov chain [14] to derive results pertaining to the probability of subfunctionalizing/pseudogenizing at (or before) the time of the *i* ^th^ mutation.

The model which we define below is motivated by the mechanics of *regulatory* subfunctionalization. Broadly speaking, subfunctionalization can occur as a non-regulatory process, however the assumptions of this model have been chosen to match the biology of regulatory subfunctionalization as closely as possible, while allowing for analysis. There is potential application of this model to other modes of subfunctionalization where the underlying mechanics are essentially similar to the mechanics of regulatory subfunctionalization. The following key assumptions should be carefully considered when applying the model to processes besides regulatory subfunctionalization:

- the process is assumed to be neutral,
- null mutations are assumed to occur independently, and at a constant rate,
- it is assumed that, due to selection pressure, an unmutated copy of each subfunction is always retained in at least one duplicate

Consider the situation described in the duplication–degeneration–complementation (DDC) process of Force et al. [5]. Immediately after some duplication event, we have two identical genes, each with *z* mutable regulatory regions. Assume the notation of Hughes and Liberles [8], taking the (Poisson) rate at which null mutations are fixed in each of the *z* mutable regulatory regions for each gene to be *u*_*r*_, and the (Poisson) rate at which null mutations fix in the coding regions for each to be *u*_*c*_.

For a fixed number *z* of the regulatory regions in the duplicate pair of genes, consider a continuous-time Markov chain {*X*(*t*),*t*≥0}, with state space

1 $$ \mathcal{A} = \{0,1,\ldots,z-1\}\cup\{S,P\},  $$

where state *i*∈{0,1,…,*z*−1} represents the number of fixed null mutations to have occurred in the case that neither subfunctionalization nor pseudogenization have happened yet, and the states *S* and *P* are introduced to represent subfunctionalization and pseudogenization respectively. *S* and *P* are both absorbing states - that is, once subfunctionalization or pseudogenization occurs, the process stops and remains in state *S* or *P*, which represent the preservation of both copies, or one copy respectively. Under the subfunctionalization process, a duplicate pair is preserved if it undergoes subfunctionalization, otherwise one gene is lost (pseudogenization) and the remaining gene is preserved.

Note that we can significantly simplify the problem by modeling the number of null mutations to have occurred in the system as whole, rather than trying to track mutations in each gene separately. As soon as a null mutation has occurred in both genes, either subfunctionalization or pseudogenization must have occurred, so we need only count the total number of mutations until one of these two possible outcomes is realized.

We define the generator for our Markov chain to be matrix **Q**= [*q*_*ij*_] where the non-zero off-diagonals are given by

2 $$ q_{{ij}} = \left\{\begin{array}{ll} 2u_{c} & \text{if } i = 0, j = P \\ 2zu_{r} & \text{if } i = 0, j = 1 \\ u_{c} & \text{if } 1 \leq i \leq z-2, j = P \\ (z-i)u_{r} & \text{if } 1 \leq i \leq z-2, j = i + 1 \text{ or } j = S \\ u_{r} + u_{c} & \text{if } i = z-1, j = P\\ u_{r} & \text{if } i = z-1, j = S. \end{array}\right.  $$

Below, we show that the rates *q*_*ij*_ in (2) are indeed the relevant transition rates by considering the evolution immediately after duplication.

**Transitions from** ***0→P*** Clearly, the process starts in state 0, since no null mutations have fixed at the instant of duplication. Null mutations fix in the coding region for each gene at a rate *u*_*c*_, and this leads to pseudogenization. Therefore transitions from 0→*P* occurs at rate 2*u*_*c*_.

**Transitions from** ***0→1*** Null mutations fix in each of the 2*z* regulatory regions at a rate *u*_*r*_, and hence transition 0→1 occurs at a rate 2*zu*_*r*_.

After the first mutation, either a null mutation fixed in one of the coding regions, and the process has been absorbed into state *P*, or a null mutation has fixed in one of the regulatory regions of one of the genes, and the process is now in state 1.

As described in [2], null mutation in the regulatory region results in the loss of some particular function for that gene, and the total loss of a function is selected against. Hence the duplicate pair must retain at least one unmutated copy of each regulatory region between them - this is the fundamental concept of subfunctionalization. It follows then that the remaining unaffected gene must be preserved, and so too must its copy of the regulatory region which has mutated in the other duplicate.

**Transitions from** ***1→P*** Since the unaffected gene now has a unique function which is protected by selective pressure, this gene is no longer susceptible to pseudogenization under the subfunctionalization process. As such, only one copy may now undergo null mutation in the coding region, which it does at a rate *u*_*c*_. Hence the rate of transitions from 1→*P* is *u*_*c*_.

**Transitions from** ***1→S*** Also, since one regulatory region in the unaffected gene is protected by selective pressure, and one region has already undergone null mutation for the other gene, each gene has *z*−1 regulatory regions which are now susceptible to null mutation. If such a mutation occurs in the previously unaffected copy, then both copies will have a unique function, and both will be protected by selective pressure. This is subfunctionalization, and hence the process transitions from 1→*S* at a rate (*z*−1)*u*_*r*_.

**Transitions from** ***1→2*** On the other hand, if a null mutation fixes in one of the *z*−1 susceptible regulatory regions of the same copy in which the previous mutation fixed then the process transitions to state 2 - as two mutations have now fixed, but the process has not yet been absorbed. Hence the process transitions from 1→2 at a rate (*z*−1)*u*_*r*_.

**Transitions from** ***i∈{1,2,..,z−2}→j*** We note that for the process to reach state *i*∈1,2,…,*z*−2 all mutations must have occurred in the regulatory regions of the same copy, since subfunctionalization (and hence absorption to *S*) occurs as soon as both copies have a unique function. Therefore, a similar argument is used to show that for all *i*∈{1,2,..,*z*−2} transitions

- from *i*→*P* occur at rate *u*_*c*_,
- from *i*→*S* occur at rate (*z*−*i*)*u*_*r*_,
- from *i*→*i*+1 occur at rate (*z*−*i*)*u*_*r*_.

**Transitions from** ***z−1→S*** When the process is in state *z*−1 there is only one regulatory region for each copy susceptible to null mutation, which occurs at a rate *u*_*r*_ for each copy. If such a mutation occurs in the so-far unaffected gene then the process transitions to state *S*, hence the rate of transition from *z*−1→*S* is *u*_*r*_.

**Transitions from** ***z−1→P*** There are two distinct ways in which the process can transition from *z*−1→*P*. The first is similar to the previous cases, with a null mutation occurring in the coding region of the copy in which all of the previous mutations have fixed, which occurs at rate *u*_*c*_. The other way is for a null mutation to fix in the last remaining regulatory region of this same gene, which occurs at a rate *u*_*r*_. Hence the rate of transition from *z*−1→*P* is *u*_*r*_+*u*_*c*_.

**Fig. 1:**
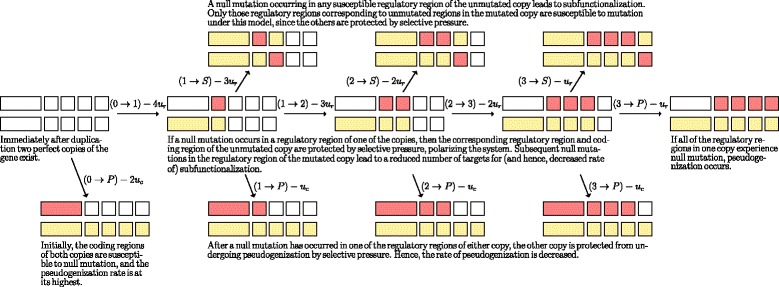
The (biological) transition diagram for *z*=4. Regions hit by null mutation are *coloured red*, and regions which are protected by selective pressure are *coloured yellow*

The full set of possible transitions for the case where *z*=4 is illustrated in Fig. 1.

#### Block matrix form of **Q**

Now, we define **Q** ^∗^ to be the block matrix of **Q** which records the transition rates between the non-absorbing states in {0,1,…,*z*−1}, and $\mathbf {V} =\ [\underline {\mathbf {v}}_{S},\underline {\mathbf {v}}_{P}]$ to be a *z*×2 matrix where (column) vectors $\underline {\mathbf {v}}_{S}$ and $\underline {\mathbf {v}}_{P}$ record the rates of transition from the non-absorbing states in {0,1,…,*z*−1} into the absorbing states *S* and *P*, respectively. We have, with **O** denoting zero matrices of appropriate sizes,

 where



This form of the generator matrix is useful, as we will make extensive use of the matrix **Q** ^∗^ and the vectors $\underline {\mathbf {v}}_{S}$ and $\underline {\mathbf {v}}_{P}$ in our derivations.

Note that since **Q** is a *transition rate* matrix, the row sums must be 0. The rows full-of-zeros corresponding to the absorbing states are interpreted as the process having zero rate of transition out from these states

## The pseudogenization cause-specific hazard rate

In this section, we give a brief overview of the pseudogenization cause-specific hazard rate. A detailed analysis of both the pseudogenization and subfunctionalization cause-specific hazard rates is given in Additional file 1: Section B. In the traditional hazard rate setting, there is only one absorbing state, and the hazard rate is a measure of the rate at which a process is absorbed, given that it has not yet been absorbed. The cause-specific hazard rate can be applied when there are several absorbing states, and is a measure of the rate at which a process is absorbed into a particular absorbing state, given that it has not yet been absorbed into any absorbing state.

Given a random variable *T* recording time until absorption, the hazard rate *λ*_*i*_(*t*), given that the process starts in state *i* is defined [14] for all *t*≥0, as

4 $${} \lambda_{i}(t)= {\lim}_{h\to 0^{+}} \frac{P(t<T<t+h|T>t,X(0) = i)}{h} = \frac{f_{i}(t)}{1-F_{i}(t)},  $$

where *f*_*i*_(*t*) is the probability density of absorption occurring at time *t* given that the process starts in state *i*, and *F*_*i*_(*t*) is the corresponding cumulative distribution function.

For the model described in the section “A continuous-time Markov chain model for Subfunctionalization” the hazard rate can be shown (see Additional file 1: Section B) to be

5 $$  \lambda_{i}(t)= \frac{-\underline{\mathbf{e}_{\mathbf{i}}}e^{\mathbf{Q}^{*}t}\mathbf{Q}^{*}\underline{1}} {\underline{\mathbf{e}_{\mathbf{i}}}e^{\mathbf{Q}^{*}t}\underline{\mathbf{1}}},  $$

where *e*_*i*_ is a (row) vector with a 1 in the *i*-th position and 0’s elsewhere, and **1** denotes a column vector of 1’s of appropriate size.

When an absorption occurs, the process transitions into an absorbing state (for the model considered here that is either *S* or *P*). This motivates the definition of the cause-specific hazard rate [15], which can be thought of as the instantaneous rate of transition into a *particular* absorbing state given that the process has not yet been absorbed. In our case, we refer to these rates as the pseudogenization cause-specific (*j*=*P*) and subfunctionalization cause-specific (*j*=*S*) hazard rates, given by

6 $${} \begin{aligned} \lambda_{ij}(t) &= {\lim}_{h\to 0^{+}} \frac{P(t<T<t+h, X(T)=j|T>t, X(0)=i)}{h}\\ &=\frac{f_{ij}(t)}{1-F_{i}(t)}\text{}, \end{aligned}  $$

After some analysis (see Additional file 1: Section B) we see that the cause-specific hazard rates for our model are given by

7 $$  \lambda_{{ij}}(t) = \frac{\left[\underline{\mathbf{e_{i}}} e^{\mathbf{Q}^{*}t}\mathbf{V}\right]_{j}} {\underline{\mathbf{e_{i}}} e^{\mathbf{Q}^{*}t}\underline{\mathbf{1}}}.  $$

Additional analysis including an investigation of the limiting behaviour, and the derivation of various measures which are not directly related to the central narrative of this paper are included in Additional file 1: Section B. Although these results are not pivotal to this work, they are likely to be of interest to more mathematically inclined readers interested in applications of the phase-type distribution to the derivation of related measures.

Now, in order to approximate the pseudogenization hazard rates, Hughes and Liberles [8] applied the following:

8 $$ {\lambda_{t}^{z}}\approx \frac{{P_{i}^{z}}}{E(\Delta T_{i})} \quad\text{for } t_{i-1}\leq t< t_{i},  $$

where the fixed points *t*_*i*_ are evaluated using

9 $$ t_{0}=0 \quad \text{and }\quad t_{i}=t_{i-1}+ E(\Delta T_{i})\quad \text{for }1\leq i\leq z.  $$

That is, the (approximating) assumption was made that the hazard rates are piece-wise constant within such specified time intervals [*t*_*i*−1_,*t*_*i*_].

However, there was an error in the approximation at which Hughes and Liberles [8] ultimately arrived. They wrote

10 $$  {\lambda_{t}^{z}} = \left\{\begin{array}{ll} 2u_{c} & \text{for } 0 \leq t < t_{1} \\ u_{c} & \text{for } t_{1} \leq t < t_{z-1} \\ u_{c} + u_{r} & \text{for } t_{z-1} \leq t < t_{z} \\ 0 & \text{for } t \geq t_{z}. \end{array}\right.  $$

Notice that no weight is given to the possibility that subfunctionalization has occurred for *t*<*t*_*z*_, and for *t*≥*t*_*z*_ no weight is given to the possibility that it has not occurred. Hence this approximation assumes that subfunctionalization occurs at the time of the *z* ^th^ mutation *t*=*t*_*z*_ exactly. This is a small but critical error, as the approximation only holds in the unlikely event that subfunctionalization occurs at time *t*=*t*_*z*_, which for most parameter sets is far from the mean time to subfunctionalization. We contend that this ultimately led to the mischaracterization of the subfunctionalization process in [8], and we will see in in the section “Shape properties of the pseudogenization rate functions” that our rate function behaves very differently to this approximation.

With a minor correction we get a good approximation for the pseudogenization cause-specific hazard rate, using the same notation we can write

11 $$ {\lambda_{t}^{z}} = \left\{\begin{array}{ll} 2u_{c} & \text{for } 0 \leq t < t_{1} \\ u_{c} & \text{for } t_{1} \leq t < t_{z-1} \\ u_{c} + u_{r} & \text{for } t \geq t_{z-1}. \\ \end{array}\right.  $$

However, as mentioned above, we contend that this is not the rate of central interest in modeling subfunctionalization.

## Pseudogenization rates

In this section, we introduce a new measure, referred to as the *pseudogenization rate*, which we contend to be of greatest interest in modeling subfunctionalization, and gene duplication more generally. This measure is a slight variation of the hazard rate which accounts for the possibility that subfunctionalization has occurred. This allows analysis of the instantaneous rate of pseudogenization when it is not possible to determine whether subfunctionalization has occurred.

Note that in the traditional hazard rate setting, all of the absorbing states are thought of as corresponding to something analogous to death, or failure. In such case, we are rarely interested in modeling the process after absorption, and hence the focus on rates in which absorption has not occurred. In contrast, the central feature subfunctionalization is the ability for both duplicates to be preserved by selective pressure when subfunctionalization occurs. Hence in our model, the absorbing state *S* corresponds to an *immunity* from subsequent failure, and as such we are interested in modeling the behaviour of the system only under the assumption that failure (i.e. pseudogenization) has not yet occurred.

Hughes and Liberles [8] assumed that subfunctionalization occurred at precisely time *t*=*t*_*z*_, and approximated the rate of pseudogenization by first approximating the cause-specific hazard rate (the hazard rate conditional on absorption into state *P*) up to time *t*=*t*_*z*_, and setting the rate to be 0 for *t*>*t*_*z*_ (see Eq. (10)). In this way, they partially overcame the fact that the cause-specific hazard rate fails to account for the primary feature of the subfunctionalization process. However, the assumption that subfunctionalization occurs at some predetermined point in time is hard to justify, and we show that there are substantial differences between the qualitative behaviour of such an approximation and the exact rate derived here.

### Pseudogenization rate for fixed *z*

Let

12 $$ T_{P}=\inf\{ t>0: X(t)=P\}  $$

be the random variable recording the time at which pseudogenization occurs. Assume that there are *z* regulatory regions in the duplicate pair of genes. We define the pseudogenization rate as

13 $$ \begin{aligned} {h^{z}_{P}}(t)&= {\lim}_{h\to 0^{+}} \frac{P(t<T_{P}<t+h|T_{P}>t,X(0)=0)}{h}\\ &= \frac{\tilde f(t)}{1-\tilde F(t)}, \end{aligned}  $$

where $\tilde F(t) = P(T_{P} \leq t)$ and $\tilde f = \tilde F'(t)$ are the corresponding cumulative distribution and probability density functions, respectively. After some analysis (see Additional file 1: Section C) this leads to a pseudogenization rate of

14 $$ {h^{z}_{P}}(t)=\frac{\underline{\mathbf{e}}_{0}e^{\mathbf{Q}^{*}t}\underline{\mathbf{v}}_{P}} {1 - \underline{\mathbf{e}}_{0}\left(e^{\mathbf{Q}^{*}t}-\mathbf{I}\right)(\mathbf{Q}^{*})^{-1}\mathbf{v}_{P}}.   $$

Given enough time, and given that pseudogenization does not occur (which we condition on in the pseudogenization rate), we would intuitively expect that subfunctionalization must eventually occur. As such, we would expect the rate ${h^{z}_{P}}(t)$ to approach zero as *t* approaches infinity, that is,

15 $$  {\lim}_{t\to\infty} {h^{z}_{P}}(t) = 0.  $$

We have confirmed this analytically in Additional file 1 Section C).

Note that it does not make sense to calculate moments of time to pseudogenization (e.g. the mean, variance) with this rate. This is because of the possible absorption into the state *S* - if subfunctionalization occurs, then the time to pseudogenization is infinite. Recalling that the pseudogenization rate does not condition on subfunctionalization not occurring, clearly then the mean time to pseudogenization is infinite. For a meaningful calculation of moments, the hazard rate should be used.

### Average pseudogenization rate (for randomly distributed *z*)

We now consider the pseudogenization rate averaged over the possible values of *z*. We let *Z* be a random variable tracking the number of regulatory regions in a duplicate pair, and define the average pseudogenization rate as

16 $$ H_{P}(t) = \sum_{z = Z_{min}}^{Z_{max}} p_{z} {h_{P}^{z}}(t).  $$

Notice that since each ${h_{P}^{z}}(t) \to 0$ as *t*→0, it follows that

17 $$ {\lim}_{t\to\infty} {H^{z}_{P}}(t) = 0.  $$

## Subfunctionalization survival function and Poisson duplication

In order to fit the model to genome data, we will make use of the survival function implied by our model together with some assumptions about the underlying gene duplication process.

By Eq. (14), the survival function corresponding to the random variable *T*_*P*_ is given by

18 $$  P(T_{P} > t) = 1 - \tilde F(t) = 1 - \underline{\mathbf{e}}_{0} \left(e^{\mathbf{Q}^{*}t}-\mathbf{I}\right) \left(\mathbf{Q}^{*}\right)^{-1}\mathbf{v}_{P}.  $$

Note that the data (handled by Hughes and Liberles [8]) contains the counts of the number of surviving duplicates at the current time. However, this number depends on the gene duplication process, which needs to be considered in the analysis. By introducing a Poisson process to model the duplication process (see Additional file 1: Section D), and a random variable *Y*(*t*) representing the number of duplicates that have survived to time *t* we are able to derive the probability that there are *y* duplicates surviving at the current time as

19 $$ P(Y(t)=y) = \frac{\left(\left(1-\tilde F(t)\right)\beta_{0}\right)^{y}}{y!}e^{-\beta_{0}\left(1-\tilde F(t)\right)},   $$

where $\tilde F(t)$ is defined in (18), and *β*_0_ is the average number of duplication events that occur in a time interval of length 0.01*s* - *s* being the expected number of substitutions per silent site. Note, the time interval is chosen as 0.01*s* since this is the size of the intervals in the data set we examine in the section “Fitting the model to genome data”.

Note that (19) defines a Poisson random variable with parameter

20 $$  \beta(t) = \beta_{0}(1-F(t)),  $$

which is the expected number of duplicates surviving to time *t* if the duplication process is Poisson with parameter *β*_0_ and the survival process is the model under discussion in this paper.

With this result, calculating the likelihood of the data given the parameters is straight-forward, and computationally tractable, as we only need to multiply over the probabilities for each of the data bins.

Next, in order to fit the parameters *β*_0_,*u*_*r*_,*u*_*c*_ and *z* to the data set, we use the maximum likelihood method with the log likelihood given by

21 $$ log(L_{\theta}) = \sum_{i} D_{i}\log(\beta(s_{i}) - \beta(s_{i}) - \Gamma\log(D_{i} + 1),  $$

where *D*_*i*_ is the count in the *i* ^th^ bin of the data set, and *s*_*i*_ is the associated cumulative number of silent substitutions per silent site, used as a proxy for time.

For the data set we examine in the section “Fitting the model to genome data”, *s*_*i*_=0.01*i*.

## Results

Here we discuss some further mathematical results which have more direct biological implications than those discussed in the methods section, as well as results from data fitting. In the section “Shape properties of the pseudogenization rate functions” we discuss results pertaining to the shape of the pseudogenization rate function introduced in the section “Pseudogenization rates”, these results have some implications about the predictions of the subfunctionalization process which are detailed further in the discussion. In the section “Fitting the model to genome data” we fit the model to the data sets of Hughes and Liberles [8] to estimate the relative rates of mutation and the number of regulatory regions comprising gene duplicates in *Homo sapiens, Mus musculus, Canis familiaris* and *Rattus norvegicus*.

## Shape properties of the pseudogenization rate functions

**Fig. 2:**
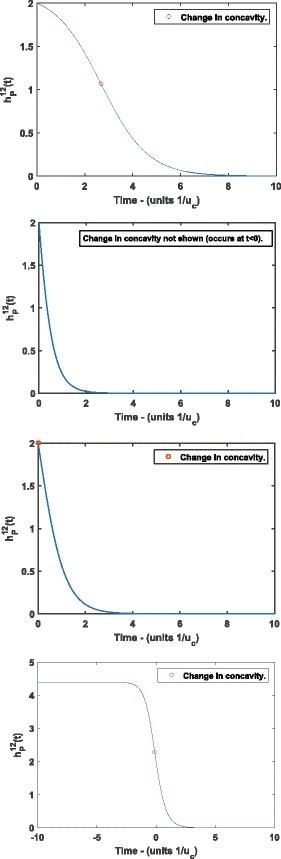
Pseudogenization rate ${h_{P}^{z}}(t)$ with for *z*=12 *γ* less than **a**, greater than **b** and equal to **c** *γ*_crit_. Panel **d** shows the overall shape of ${h_{P}^{z}}(t)$, with negative values of *t* included. **a** Pseudogenization rate ${h_{P}^{z}}(t)$ with $\gamma < \gamma _{\text {crit}}^{z}$. The change in concavity occurs at *t*≈2.7. As such, the sigmoidal nature of the function is apparent - we see an initially slowly decreasing hazard rate which decreases more and more rapidly up to the change in concavity, after which the decline in the hazard rate slows, and approaches the asymptote at zero. **b** Pseudogenization rate ${h_{P}^{z}}(t)$ with $\gamma > \gamma _{\text {crit}}^{z}$. Here the change in concavity occurs for some *t*<0, and hence cannot be seen in real, physical time (*t*>0). The shape is not obviously sigmoidal, and looks similar to that of an exponential decay. The rate of pseudogenization is initially declining rapidly, before approaching its asymptote at zero. **c** Pseudogenization rate ${h_{P}^{z}}(t)$ with $\gamma = \gamma _{\text {crit}}^{z}$. Here the change in concavity occurs at exactly *t*=0. This is qualitatively similar to the case in panel **b**, with the pseudogenization rate rapidly declining, and the decline becoming slower as the rate approaches its asymptote at zero. **d** Pseudogenization rate ${h_{P}^{z}}(t)$ taken as a function over all $\mathbb {R}$. This gives a complete picture of the shape of the pseudogenization rate function. Smaller values of *γ* translate the graph to the right, and result in a longer initial period of slowly declining pseudogenization rate. If *γ*>*γ*_crit_, the point of inflection occurs to the left of *t*=0, and we see behaviour similar to panel **a**

In this section we analyze the shape properties of the pseudogenization rate function, and consider the implications of these properties on the predictions of the subfunctionalization process. We show that our model predicts a sigmoidal shaped pseudogenization rate, we also show this does not always imply the broadly concave decreasing hazard rate (where the instantaneous rate of pseudogenization decreases faster and faster as time elapses) suggested by Hughes and Liberles [8]. We find the critical value at which the function’s behaviour shifts from being obviously sigmoidal to apparently exponential (i.e. convex decreasing), which we illustrate in Fig. 2.

Hughes and Liberles [8] found that a hazard rate following a Weibull distribution provided an extremely good fit to gene duplicate data. Konrad et al. [12] fit sigmoid (S-shaped) curves to their data sets. Panel (d) of Fig. 2 shows that this is indeed the shape arising from our model. Note that the time axis in Panel (d) of Fig. 2 includes negative values. There is an obvious continuation of ${h_{P}^{z}}(t)$ to the domain $t \in \mathbb {R}$ (i.e. including negative *t*) where we take the same expression for ${h_{P}^{z}}$ without interpreting *t* as corresponding to time.

Considering the behaviour of ${h_{P}^{z}}(t)$ for negative *t* can be useful, as an important qualitative feature is whether the change of concavity occurs in *t*≥0, or *t*<0. At the point of inflection, the function changes between decreasing more and more quickly, and decreasing more and more slowly. As far as our biological interpretation of ${h_{P}^{z}}(t)$ (as the rate of pseudogenization) is concerned, we are only interested in *t*≥0. Hence, whether this change of behaviour occurs in real, physical, time (*t*≥0) or not is an important defining feature of the characteristics of the process. This is demonstrated in Fig. 2.

Notice that there are two distinct predictions for the behaviour of this process. The process can begin with a rapid decline in the rate of pseudogenization, quickly approaching its asymptote at zero, and leaving relatively little opportunity for pseudogenization to occur. Alternatively, it can start with a slowly declining pseudogenization rate, giving more time for pseudogenization to occur before a high probability of subfunctionalization eventually takes over and the rate of pseudogenization begins to rapidly decline towards it’s aymptote at zero.

In the absence of a relative clock (such as synonymous site mutations, which we use in the section “Fitting the model to genome data”) the parameters *u*_*c*_ and *u*_*r*_ are only meaningful relative to each other. As such, non-dimensionalization is a convenient technique to reduce the number of parameters from 3 to 2.

We can replace parameters *u*_*c*_ and *u*_*r*_ with their ratio *γ*=*u*_*r*_/*u*_*c*_, and work with time units 1/*u*_*c*_. Thus *u*_*c*_ can be thought of as the units of the rate (note that the rate units *u*_*c*_, are the inverse of the time units 1/*u*_*c*_ as usual). This technique is applied implicitly throughout many of the results that follow.

**Fig. 3:**
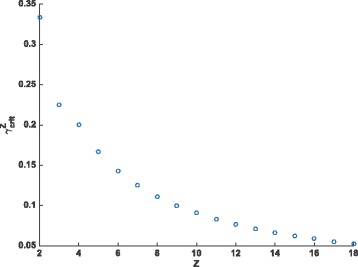
Critical values $\gamma _{\text {crit}}^{z}$ for various values of *z*. When $u_{r}/u_{c} \leq \gamma _{\text {crit}}^{z}$ the change in concavity for the pseudogenization rate function will occur in positive time. Otherwise, the change occurs in negative t and the sigmoidal nature of the function will not be apparent when plotted for *t*>0

The change in concavity will occur when the second derivative is zero, i.e. when $h_{P}^{z\prime \prime }(t) = 0$. We define $\gamma _{\text {crit}}^{z}$ to be the ratio *u*_*r*_/*u*_*c*_ at which the second derivative is zero precisely when *t* is zero, i.e the ratio *u*_*r*_/*u*_*c*_ such that $h_{P}^{z\prime \prime }(0) = 0$. When $ 0 < \gamma = u_{r}/u_{c} < \gamma _{\text {crit}}^{z}$ the concavity of ${h_{P}^{z}}(t)$ will change for some *t* ^∗^>0, and we see the behaviour where an initially slowly-declining hazard rate decreases more and more quickly before slowing back down as it approaches zero. Otherwise, the change in concavity occurs for some negative value of *t*, and in this case the hazard rate begins its rapid decline immediately, with the rate of decline slowing from it’s initially-high value at all times. Figure 3 shows the values of $\gamma _{\text {crit}}^{z}$ for various values of *z*.

The sigmoidal shape is in good qualitative agreement with previous results, and in Additional file 1: Section E we retro-fit our model to existing phenomenological approximations due to Teufel et al. [11] and Konrad et al. [12], finding particularly good agreement with the shape of the hazard function in Teufel et al. [11]. The continuation of ${h_{P}^{z}}(t)$ to include negative *t* is particularly useful in this analysis, and in Additional file 1: Section G we prove a result about the limit as *t*→−*∞* of a generalization of ${h_{P}^{z}}(t)$ applicable to any absorbing CTMC with a finite state space.

We note that the shape of ${h_{P}^{z}}(t)$ is only sigmoidal for 0<*u*_*r*_<*u*_*c*_. For *u*_*r*_=0 the shape of ${h_{P}^{z}}(t)$ becomes exponential, and when *u*_*r*_>*u*_*c*_, ${h_{P}^{z}}(t)$ may have multiple turning points, or points of inflection. However, these are not realistic parameter sets for subfunctionalization.

### **Example 4.1**

In this example, we examine the shape of the pseudogenization rate for *z*=12, and $\gamma = u_{r}/u_{c} = 0.005 < \gamma _{\text {crit}}^{12} = 0.0714$. In this case, the sigmoidal shape of the rate function is quite apparent. This is because the change in concavity occurs in physical time, and hence in Panel (a) of Fig. 2 we can see that the rate is relatively flat near *t*=0.

### **Example 4.2**

For this example, we examine the shape of the pseudogenization rate for *z*=12 and $\gamma = u_{r}/u_{c} = 0.0714 = \gamma _{\text {crit}}^{12}$, shown in Panel (c) of Fig. 2. Here we see little evidence of the sigmoidal shape of the rate function for *t*≥0, which could be well approximated by an exponential decay.

### **Example 4.3**

In this example, we examine the shape of the pseudogenization rate for *z*=12 and $\gamma = u_{r}/u_{c} = 0.2 > \gamma _{\text {crit}}^{12} = 0.0714$. In this case, the point of inflection does not occur in physical time and the shape of the function becomes indistinguishable from an exponential decay. Panel (b) of Fig. 2 shows the rate function for this example.

## Fitting the model to genome data

In this section we fit the model to the data set analyzed in Hughes and Liberles [8], the data set is included in Additional file 2, while the MATLAB scripts used for analysis are in Additional file 3. This data consists of counts of the number of duplicate pairs in several genomes with corresponding estimates of the cumulative number of silent substitutions per silent site, binned in intervals of length 0.01*s*, where *s* is the cumulative number of silent substitutions per silent site. The silent substitutions can be used as a proxy for time, and the intervals of length 0.01*s* represent on average 1.1 million years. Hughes and Liberles [8] tested the quality of the alignments by comparing the mean and median fraction of alignment columns which were gap free. They concluded that the alignments for the four species *M. musculus, R. norvegicus, H. sapiens and C. familiaris* were of high quality, and these are the data sets we will examine here. In [8] Hughes and Liberles fit off-the-shelf survival functions to these data sets. Here we assume that the underlying duplicate process is a Poisson process, and fit the survival function derived from our model as discussed in the section “Subfunctionalization survival function and Poisson duplication” of the Methods.

We computed maximum likelihood estimates (MLEs) $\hat \theta _{z} =\ [\hat u_{r}, \hat u_{c}, \hat \beta _{0}]$ for *u*_*r*_,*u*_*c*_ and *β*_0_ for each *z* from 2 to 20 for four mammalian genomes. We call the best of these *z*’s (in terms of likelihood) $\hat z$, with the understanding that this is not a true maximum likelihood estimate, since we restricted $\hat z \in \{2,3,\ldots,20\}$. We chose this truncation because it is unlikely that a gene would have in excess of 20 regulatory regions [8]. The case *z*=1 is excluded, as subfunctionalization cannot occur in this case, and the survival model reduces to an exponential survival function with parameter 2(*u*_*c*_+*u*_*r*_) when *z*=1 or *u*_*r*_=0.

**Fig. 4:**
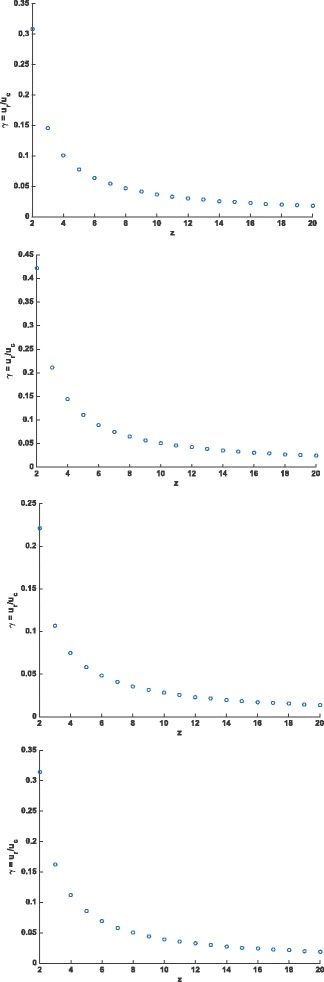
Maximum likelihood estimates for *γ*=*u*_*r*_/*u*_*c*_ for each *z*=2,3,…,20 for four mammalian species. **a** *γ* vs *z* in the MLE for *Canis familiaris*. **b** *γ* vs *z* in the MLE for *Homo sapiens* **c** *γ* vs *z* in the MLE for *Rattus norvegicus* **d** *γ* vs *z* in the MLE for *Mus musculus*

The ratio *γ*=*u*_*r*_/*u*_*c*_ and *z* appear to be strongly correlated in the MLEs, as shown in Fig. 4. A power law relation between *γ* and *z* appeared to fit quite well, with *R* ^2^ values >0.97 for each of the four genomes.

We compared the fit of our survival function (18) against Weibull and exponential functions using relative likelihood via the AIC [16]. For all four genomes, our model outperformed the exponential function, but was itself outperformed by the Weibull function in the rat, mouse and human genomes. In the canine genome there was insufficient evidence to choose between the Weibull function and the survival function derived from the model.

Mechanistic models can contain parameters that are part of the generative process but add little to data fitting, sometimes resulting in less support for mechanistic models when compared to simpler models, even when the mechanistic models give more accurate inference of the underlying process as judged by the accuracy of parametrization (see Liberles et al. [17]). With this justification in mind, we move forward with analysis of the results of fitting the mechanistic subfunctionalization model to genomic data. The analysis of mechanistic parameters is conditional on the generative process being what is modeled, namely subfunctionalization.

**Fig. 5:**
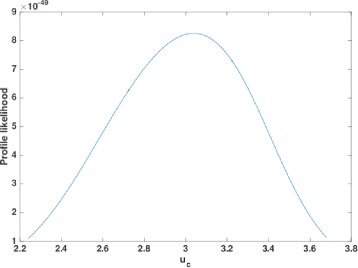
The profile likelihood curve for *u*_*c*_ in the *Rattus norvegicus* genome

To estimate the relative rates of mutation *γ*=*u*_*r*_/*u*_*c*_ together with the mean number of duplicates per 0.01*s*, *β*_0_, we fixed $z = \hat z$ and computed *e* ^2^ likelihood intervals for each of the parameters using the profile likelihood approach. We also calculated *e* ^2^ likelihood intervals for *z* using the values of the MLE. In the regular asymptotic case *e* ^2^ likelihood intervals are equivalent to 95.4*%* confidence intervals [18]. Since the shape of the profile likelihood is quite standard (for example, see Fig. 5), it is reasonable to regard these intervals as approximate 95% confidence intervals. These results are summarized in Table 1.

**Table 1:** *e* ^2^ likelihood intervals and maximum likelihood estimates for four species

	lower	MLE	upper	lower	MLE	upper
	*Rattus norvegicus*			*Mus musculus*		
*u*_*c*_	2.24	3.04	3.68	18.03	20.07	22.33
*u*_*r*_	0.00	0.67	2.41	2.87	3.26	3.69
*β*_0_	186.63	204.04	221.06	633.00	680.84	731.62
*z*	2	2	20	3	3	5
	*Homo sapiens*			*Canis familiaris*		
*u*_*c*_	12.44	14.71	17.43	6.36	7.74	9.25
*u*_*r*_	2.52	3.11	3.80	1.30	2.39	3.45
*β*_0_	315.55	348.11	384.05	114.12	129.07	145.77
*z*	3	3	5	2	2	20

There were some minor identifiability issues in fitting the model to this data, in particular, for the *Rattus norvegicus* and *Canis familiaris* genomes, we were able to find good relative likelihood scores for any *z*=2,3,…20, which prevents us from reliably estimating *z* for these two genomes. Together with the close correlation between *z* and *γ*=*u*_*r*_/*u*_*c*_ this could be overcome by fixing one or more of the parameters using some outside analysis. In both cases, the maximum likelihood estimate for *z* was $\hat z = 2$.

As a point of interest, we ran some simulations using parameters similar to those estimated for the rat genome. We simulated bins of data identical to those in the data, i.e. 30 bins corresponding to 0.01*s*,0.02*s*,…0.3*s*, and found that the parameters of the model were difficult to recover in this case, with the MLE value for *z* varying between runs with the same parameters. In some runs, even when *z* was fixed to the correct value used in the simulation *u*_*r*_ and *u*_*c*_ were not able to be accurately recovered, with *γ*=*u*_*r*_/*u*_*c*_ varying from around 0.05 to 0.3 (true value 0.2) in the handful of simulations we ran. The true parameters fell within the *e* ^2^ likelihood intervals, but it wasn’t until we increased the number of intervals to 100 that we started to get more reliable recovery of the simulation parameters.

The simulation analysis provided some insight into the relatively unstable results for the rat and dog genomes. We suspect that the combination of low count values (and hence low *β*_0_), together with the relatively low estimated mutation rate, leads to an overall lack of information in the data for these two genomes compared to the others, and hence the difficulty pinpointing parameters. Based on the results of the simulations we ran, we expect that the likelihood intervals for these genomes are somewhat reliable, while the maximum likelihood estimates themselves are probably not very precise.

We also note that for the rat genome the value *u*_*r*_=0 was within the established likelihood intervals. In this case, the survival function for our model reduces to an exponential survival function with parameter 2*u*_*c*_. The lack of differentiation between the mechanistic and phenomenological models in this case is likely due to the previously mentioned relative lack of information in the rat data.

For the *Homo sapiens* and *Mus musculus* genomes the maximum likelihood estimate for *z* was $\hat z = 3$ in both cases, with *z*=3,4,5 falling in the *e* ^2^ likelihood interval. In these two cases the higher mutation rate estimates, together with larger counts of duplicates are suggestive of more informative data, and the results are in-line with this suggestion. We expect the maximum likelihood parameter estimates to be more reliable in these cases.

Note that we model the evolution of a *pair of gene duplicates*, and thus our estimates implicitly assume that all of the duplicates in the genomes analyzed have the same parameters as each other. That is, the maximum likelihood estimate $\hat z$ is an estimate for the number of regulatory regions each gene has assuming they all have the same number of regulatory regions. Similarly, the estimates for *u*_*c*_ and *u*_*r*_ assume a consistent rate of mutation throughout all of the genes in the data set.

These assumptions are inherent to the application of models at the level of individuals (or in this case, pairs of individuals) to larger data sets, however the importance of these assumptions is particularly apparent when considering fixed parameters such as *z*. In the absence of parameter *z*, we could think of the Poisson rates *u*_*c*_ and *u*_*r*_ as measuring an average mutation rate across the genome, however, since *z* is fixed there is no similar interpretation for the number of regulatory regions. With this in mind, we can think of *u*_*c*_ and *u*_*r*_ as average mutation rates given that all duplicates examined have exactly *z* regulatory regions.

In order to relax this assumption, we computed analogous maximum likelihood estimates for randomly distributed *Z* using a truncated (2≤*Z*≤20) Poisson distribution with parameter *α*, given by

22 $$ P(Z = z) = \frac{\alpha^{z}}{z!}e^{-\alpha}\left(\sum_{k = 2}^{20}\frac{\alpha^{k}}{k!}e^{-\alpha} \right)^{-1},  $$

which resulted in distributions where the majority of the weight was at the lower end of the truncation, *Z*=2. However, this result should be viewed with care, as the procedure is biased in favour of results which place the majority of the weight around the points of truncation, *Z*=2 and *Z*=20. This is because having the majority of the weight on a single value of *Z* allows for the parameters *β*_0_,*u*_*r*_,*u*_*c*_ to be chosen so as to best fit the particular value of *Z*, giving a distinct advantage over more evenly weighted distributions.

It should be noted that this analysis relies upon a particular set of assumptions, that the mode of action on all genes in the genome is subfunctionalization of the regulatory regions. It should be further noted that subfunctionalization of coding sequences is possible, but in some circumstances may not be a neutral process characterized by the same dynamics. For example, subfunctionalization from a ligand-binding generalist to a pair of specialists that are specific to an individual or set of ligands might require selection to attain that specificity (see Liberles et al. [19] for further discussion of this point). Other types of coding sequence subfunctionalization, like regulation mediated by post-translational modification of specific amino acids might occur with regulatory region-like dynamics.

## Discussion

The present work partially contradicts Hughes and Liberles [8] characterization of subfunctionalization by an initially constant, and then broadly decreasing, concave hazard function which has been adapted by subsequent works [10–12]. The intuition behind this characterization can be explained by thinking of the initial period of constant hazard rate as corresponding to the waiting time for the first mutation. After this first mutation the unaffected gene will be selectively protected against pseudogenization, and hence there is a sharp decline in the hazard rate (from 2*u*_*c*_ to *u*_*c*_ in terms of both the model discussed in [8] and the model discussed in this paper). Once the first mutation has fixed, there is now an opportunity for an additional mutation to lead to subfunctionalization, in which case the rate of pseudogenization will decrease from *u*_*c*_ to 0. The probability that subfunctionalization occurs before time *t* is rapidly increasing with *t*, and this leads to the concave decline in the hazard function.

In contrast, our hazard rate ${h_{P}^{z}}(t)$ has a sigmoid shape, which includes a period of concave decline, followed by a change in concavity, and a period of convex decline. Considered as a function of *t*>0, ${h_{P}^{z}}(t)$ can include short or long initial periods of concave decline, or no period of concave decline at all, depending on the ratio *γ*=*u*_*r*_/*u*_*c*_. In all cases the hazard rate ${h_{P}^{z}}(t)$ will be declining convexly towards 0 from some point *t*=*t*_crit_ onwards.

For $\gamma > \gamma _{\text {crit}}^{z}$ the change in concavity occurs for some *t*>0, and we see a short or long period of concave decline followed by convex decline (see Panel (a) of Fig. 2). This essentially agrees with the characterization of Hughes and Liberles [8]. In fact, Fig. 7 of [8] shows a period of convex decline in the mean hazard rate when averaging over certain distributions of the number of regulatory regions *z*. However, the present work shows that even with a fixed number of regulatory regions *z* a change in concavity will occur in the hazard rate ${h_{P}^{z}}(t)$. This suggests that the period of convex decline is more fundamental to subfunctionalization than suggested by the characterization of [8], which focused particularly on the period of concave decline.

For $\gamma < \gamma _{\text {crit}}^{z}$ the difference in our hazard rate and the characterization of Hughes and Liberles [8] is more stark, and warrants a careful reconsideration of the biological intuition. In this case, the hazard rate ${h_{P}^{z}}(t)$ is convexly decreasing for all *t*>0, much like an exponential decay (see Panel (b) of Fig. 2).

This convexly decreasing hazard rate ${h_{P}^{z}}(t)$, as seen in our model, contradicts the characterization of subfunctionalization as predicting a concave decline in the hazard rate. However, the prediction of a convex decay comes from the same mechanics which motivate, and (for certain parameter sets) give rise to the concave characterization. Thus, we suggest some new intuition for duplicates that have a large nonfunctionalizing mutation rate in the regulatory regions.

Noting that ${h_{P}^{z}}(t) = {\lambda _{P}^{z}}(t)P(X(t) \neq S)$, consider now the evolution of a duplicate pair with some large nonfunctionalizing mutation rate in the regulatory regions, relative to that in the coding regions. Two important features are then apparent which explain the convex decline of the hazard rate ${h_{P}^{z}}(t)$.

First, there is a high probability that an initial nonfunctionalizing mutation in the regulatory region occurs in a short time. This results in an initial, rapid decrease in the rate of pseudogenization, since the first nonfunctionalizing change in a regulatory region results in a change in the pseudogenization rate from 2*u*_*c*_ to *u*_*c*_.

Second, once this first mutation has fixed, there is a very high rate of subfunctionalization (rate of transition to *S*). Since ${h_{P}^{z}}(t) = {\lambda _{P}^{z}}(t)P(X(t) \neq S)$, the term *P*(*X*(*t*)≠*S*) (which is decreasing exponentially) dominates. Together with the initially rapidly decreasing pseudogenization, this leads to the exponential-like decay of ${h_{P}^{z}}(t)$.

Biologically speaking, the case $\gamma < \gamma _{\text {crit}}^{z}$ could correspond to a set of genes with complex regulation and a small coding sequence target for both nonfunctionalization and for the accumulation of synonymous mutations. This analysis predicts that genes with the features of complex regulation requiring multiple functional transcription factors that have the ability to be subfunctionalized together with a short coding sequence would be strong candidates for subfunctionalization rather than nonfunctionalization, and would be less likely to be characterized by a concave hazard function. These conditions could be met, for example, when genes are expressed in multiple tissues at different levels. When examined in actual genomic data, genomes dominated by such genes have not been observed, but represent a theoretical possibility in which subfunctionalization would be better characterized by a convex than a concave declining hazard function.

At the individual gene level, there are several classes of proteins that might be thought of as candidates for falling into this space. Casein is a longer protein that could accumulate synonymous changes, but would be hard to nonfunctionalize. It is expressed in multiple tissues, but the regulation of its expression and the strength of negative selection on each regulatory domain is not well known [20].

Another example of genes that might fall into this category are hormones like insulin and gonadotropin hormone releasing hormone (GnRH) that are relatively short proteins, although they are less broadly expressed [20]. GnRH has in fact been retained after multiple gene duplication events in vertebrate lineages with functional divergence between copies (the functions in the ancestral state are not known) [21].

Our final example of genes that are candidates to show this behaviour are the intrinsically disordered proteins, which are shorter than folded proteins on average, and may be more mutationally robust to nonfunctionalizing mutations [22]. What is unclear at this stage is the selection on their function and their expression.

While these types of genes are not likely to dominate any whole genome analysis, the model predicts that genes with small mutational footprints for nonfunctionalizing mutations and large footprints for regulatory subfunctionalization would undergo subfunctionalization at high rates.

## Conclusions

In this paper, we have introduced a new mathematical model for the biological process of subfunctionalization. The model is mechanistically motivated, and analytically and computationally tractable. We have analyzed the model in detail, deriving key performance measures including mean times to subfunftionalization/pseudogenization, variances, and hazard functions. The parameters of the model correspond to real biological processes, and as such we have been able to use model fitting to make some inferences about the nature of gene duplicates in four mammalian genomes. The model provides a means to calculate exact results pertaining to subfunctionalization under different rates of mutation for fixed or randomly distributed numbers of regulatory regions, and to estimate the values of these parameters via maximum likelihood.

This gives a fresh perspective on the subfunctionalization process, and results from the mathematical analysis show that the subfunctionalization model predicts behaviour which is qualitatively more in-line with empirical reality than previously thought. The model fits well to empirical data sets, with its survival function providing a better fit than the often-used exponential function in all of the cases examined. The highly flexible Weibull distribution outperformed our model in three of the four cases examined in terms of absolute fit, however the main advantage of a mechanistic model over a flexible phenomenological model is the fact that it uses biologically meaningful parameters.

Our analysis of the four mammalian genomes (using data handled by Hughes and Liberles [8]) suggests that (subject to our modeling assumptions) gene duplicates most likely have only a few regulatory regions, and that the rate of mutation in these regulatory regions is around 5–10 times smaller than the rate of mutation in the coding region, which is suggestive of the relative mutational target sizes. This is the first model-based estimate of the number of regulatory regions in gene duplicates. The estimates, based upon an assumption of duplicate gene preservation through the subfunctionalization process are in-line with the conventional thinking. Force et al. [5] suggested that the ratio of mutations be about 0.1, and Hughes and Liberles [8] suggested that between 2 and 12 regulatory regions were realistic. Mechanistic characterization of mutational potentials in protein-coding genes from molecular-level analysis can add additional insight into these parameterizations.

Further, we have discovered a previously unrecognized characterization of subfunctionalization which could theoretically occur in genes with small mutational footprints for nonfunctionalizing mutations and large footprints for regulatory subfunctionalization. While these genes are not likely to dominate any whole genome analysis, the model predicts that they would undergo subfunctionalization at high rates, with a sharply convex declining hazard rate for pseudogenization. This contrasts the characterization of Hughes and Liberles [8], which features a (primarily) concave declining hazard rate and is confirmed by our model for genes with larger footprints for nonfunctionalization mutations.

In order to make accurate inferences in a phylogenetic context, increasingly realistic models of the underlying evolutionary processes will be required. While the model described here tackles only a small piece of the overall picture of the evolution of gene duplicates, we believe it is helpful towards the development of a mechanistic model for the overall process of gene duplicate evolution. Our model describes the evolution of a pair of duplicates evolving under subfunctionalization with relatively few assumptions. In terms of modeling this process, the main weaknesses of the model are the lack of detailed modeling of the coding region, which is treated as a single unit which can undergo nonfunctionalizing mutation at a constant Poisson rate, and the assumption of constant Poisson rates of mutation, consistent across the regulatory regions.

Applying this model to whole-genome data, as in the section “Fitting the model to genome data”, requires a broadening of scope beyond the evolution of a pair of duplicates, and beyond the process of subfunctionalization. We have implicitly assumed that all of the duplicates in each of the genomes examined are evolving under subfunctionalization, which is not likely to be a realistic assumption. Also, we have fit a single parameterization of the model to each genome. This is similarly unrealistic, as it would imply that all duplicates have the same number of regulatory regions as each other, and that all of these regulatory regions undergo nonfunctionalization at the same rate. At the whole-genome level a more realistic approach would be to allow each duplicate pair to evolve according to a separate parameterization of the model, however this method would have no statistical power.

Furthermore, some attributes of the generative process have not been modeled. One example is the underlying population genetic process of fixation of both the gene duplicate itself and mutations in the gene. When these mutational events are non-neutral, this process becomes particularly difficult to model. Similarly, the organisms studied here at genomic levels are diploid, but no model for dominance and the underlying genetics has been proposed. Finally, a steady state mutational process has been assumed, with a constant duplication rate.

Two distinct avenues for extension of the work presented here are apparent. The first is in widening the scope of the model itself, for example by including other processes besides subfunctionalization, such as neofunctionalization, dosage balance, and potentially other processes described in Innan and Kondrashov [3] to create a more complete model for the evolution of gene duplicates. The second avenue for extension would focus on the application of models in broader analysis. Here we applied our model in a whole-genome analysis to get some estimates of mutation rates and number of regulatory regions. The larger problems of the inference of parameters in a phylogenetic context, gene tree/species tree reconciliation, and ancestral copy number inference from multi-species data in a phylogenetic context are of particular interest.

## Additional files

Additional file 1 Contains most of the mathematical details supporting this analysis, including derivations and proofs of various results referenced throughout the main body of this work. (PDF 329 kb)

Additional file 2 Contains the data set analyzed in this work. The data was originally published and analyzed by Hughes and Liblerles [8]. (ZIP 120 kb)

Additional file 3 Contains MATLAB scripts used in this analysis, together with some directions for use. (ZIP 6 kb)

## Abbreviations

CTMC: Continuous time Markov chain
DDC: Duplication–degeneration–complementation
GnRH: Gonadotropin hormone releasing hormone
MLE: Maximum likelihood estimate
