# Supplementary material for: Analysis of a mechanistic Markov model for gene duplicates evolving under subfunctionalization
Source: BMC Evol Biol. 2017 Jan 31;17:38. doi: 10.1186/s12862-016-0848-0 (PMC5282866; doi:10.1186/s12862-016-0848-0)
Supplement: Additional file 1 — Contains most of the mathematical details supporting this analysis, including derivations and proofs of various results referenced throughout the main body of this work. (PDF 329 kb) [file 12862_2016_848_MOESM1_ESM.pdf]

Appendices
Analysis of a mechanistic Markov model for gene duplicates evolving under subfunctionalization.

Tristan L. Stark, David A. Liberles, Barbara R. Holland, Malgorzata M. O'Reilly

A Probabilities corresponding to the i-th mutational events

Here we derive probabilities P_i^{*z} and S_i^{*z} corresponding to the i-th mutational events at which absorption into either pseudogenization or subfunctionalization (as described in the Background section) occurs, respectively. We note that, although these probabilities are not used in the main body of the work, they are useful measures of interest in their own right. Some of these results have previously been discussed by Force et al [5], here we will derive them from a Markov chain analysis.

We consider the situation described in the duplication–degeneration–complementation (DDC) model of Force et al [5]. Immediately after some duplication event, we have two identical genes, each with z mutable regulatory regions. We use the notation of Hughes and Liberles [8], taking the (Poisson) rate at which null mutations are fixed in each of the z mutable regulatory regions for each gene to be u_r, and the (Poisson) rate at which null mutations fix in the coding regions for each to be u_c.

For a fixed number z of the regulatory regions in the duplicate pair of genes, consider a discrete-time Markov chain $\{X_n, n = 1, 2, \ldots\}$, whose states are observed at the n-th mutational events n, with state space

$$\mathcal{A} = \{0, 1, ..., z-1\} \cup \{S, P\}, \tag{27}$$

where state $i \in \{0, 1, ..., z-1\}$ represents the number of fixed null mutations to have occurred in the case that neither subfunctionalization nor pseudogenization have happened yet, and the states S and P are introduced to represent subfunctionalization and pseudogenization respectively. S and P are both absorbing states, since under the subfunctionalization model a duplicate pair is preserved if it undergoes subfunctionalization, otherwise one gene is lost (pseudogenization) and the remaining gene is preserved. Note that this is the embedded chain [14] of the continuous-time Markov chain that we derive in Section 1.

We assume that the initial state at time zero is $X_0 = 0$. For $i = 1, \ldots, z$, define

- $P_i^z = P(X_i = P | X_k \notin \{S, P\}, k = 1, \ldots, i-1) = P(X_i = P | X_{i-1} = z-i+1)$, interpreted as the probability of pseudogenizing at the i-th mutational event, given that neither pseudogenization nor subfunctionalization has occurred yet,

- $S_i^z = P(X_i = S | X_k \notin \{S, P\}, k = 1, \ldots, i-1) = P(X_i = S | X_{i-1} = z - i + 1)$, interpreted as the probability of subfunctionalizing at the i-th mutational event, given that neither pseudogenization nor subfunctionalization has occurred yet,

- $P_i^{*z} = P(X_i = P, X_k \neq P, k = 1, \ldots, i-1)$, interpreted as the probability of pseudogenizing at the time of the i-th mutational event,

- $S_i^{*z} = P(X_i = S, X_k \neq S, k = 1, \ldots, i-1)$, interpreted as the probability of subfunctionalizing at the time of the i-th mutational event.

By the analysis of the Markov chain, with P_i^z evaluated as in [8], we have $S_1^z = 0$,

$$P_1^z = \frac{u_c}{u_c + z u_r}, \tag{28}$$

$$P_z^z = \frac{u_c + u_r}{u_c + 2u_r}, \tag{29}$$

and, for $2 \leq i \leq z-1$,

$$P_i^z = \frac{u_c}{u_c + 2(z-i+1)u_r}; \tag{30}$$

for $2 \leq i \leq z$,

$$S_i^z = \frac{(z-i+1)u_r}{u_c + 2(z-i+1)u_r}; \tag{31}$$

for $1 \leq i \leq z$,

$$P_i^{*z} = \prod_{k=1}^{i-1} (1 - P_k^z - S_k^z) \cdot P_i^z \tag{32}$$

and

$$P_{i+1}^{*z} = (1 - P_i^z - S_i^z) \cdot \frac{P_{i+1}^z}{P_i^z} \cdot P_i^{*z}; \tag{33}$$

for $3 \leq i \leq z$,

$$S_i^{*z} = \prod_{k=1}^{i-1} (1 - P_k^z - S_k^z) \cdot S_i^z \tag{34}$$

and

$$S_{i+1}^{*z} = (1 - P_i^z - S_i^z) \cdot \frac{S_{i+1}^z}{S_i^z} \cdot S_i^{*z}; \tag{35}$$

and also $S_1^{*z} = 0$, $S_2^{*z} = (1 - P_1^z - S_1^z) \cdot S_2^z$.

Clearly, since absorption into $\{S, P\}$ must occur by the time of the z-th mutational event with probability 1, we also have

$$\sum_{i=1}^{z} (P_i^{*z} + S_i^{*z}) = 1, \tag{36}$$

and note that the quantity F_i^{*z} defined as

$$F_i^{*z} = \sum_{k=1}^{i} (P_k^{*z} + S_k^{*z}), \tag{37}$$

is the probability of having been absorbed into $\{S, P\}$ at or before the time of the i-th mutational event.

Further, denote by T_i the random variable recording the time of the i-th mutational event, let $\Delta T_i = T_i - T_{i-1}$, and note that, as in [8],

$$E(\Delta T_i) = \begin{cases} \frac{1}{u_c + 2(z-i)u_r} & \text{if } 1 \leq i \leq z, \\ \frac{1}{2(u_c + zu_r)} & \text{if } i = 1, \\ \frac{1}{u_c + 2u_r} & \text{if } i = z. \end{cases} \tag{38}$$

B Hazard rates conditional on not having been absorbed into $\{S, P\}$

Here we consider the hazard rates, cause-specific hazard rates and related measures pertaining to the CTMC defined in Section 1. These results are briefly mentioned in Section 2, however a detailed analysis is performed in this appendix.

Recall equation (4) from Section 2 the definition of the hazard rate,

$$\lambda_i(t) = \lim_{h \to 0^+} \frac{P(t < T < t + h | T > t, X(0) = i)}{h} = \frac{f_i(t)}{1 - F_i(t)}, \tag{4}$$

The analysis here is based on the results for phase-type distributions [13].

B.1 Hazard rate (absorption into $\{S, P\}$)

Let

$$T_{\{S,P\}} = \inf\{t > 0 : X(t) \in \{S, P\}\} \tag{39}$$

be the time at which the absorption into $\{S, P\}$ occurs. Following the definition in (4), the hazard rate at time t for absorption into $\{S, P\}$, given that the process starts in state $i \in \{0, 1, \ldots, z - 1\}$, is given by

$$\lambda_i(t) = \lim_{h \to 0^+} \frac{P(t < T_{\{S,P\}} < t + h | T_{\{S,P\}} > t, X(0) = i)}{h} = \frac{f_i(t)}{1 - F_i(t)}, \quad (40)$$

where $f_i(t)$ is the probability density of absorption occurring at time t given start in state i, and

$$F_i(t) = \int_{u=0}^{t} f_i(u) du \quad (41)$$

is the corresponding cumulative distribution function. The rate (40) measures the instantaneous rate of absorption into any absorbing state, given that the process has not yet been absorbed. That is the *hazard rate corresponding to absorption into* $\{S, P\}$.

From the analysis of the Markov chain and the results in [16, 17], it follows that

$$\begin{aligned} f_i(t) &= \underline{\mathbf{e_i}} e^{\mathbf{Q}^* t} \mathbf{V} \underline{\mathbf{1}} \\ &= -\underline{\mathbf{e_i}} e^{\mathbf{Q}^* t} \mathbf{Q}^* \underline{\mathbf{1}}, \end{aligned} \quad (42)$$

where $\mathbf{e_i}$ is a (row) vector with a 1 in the i-th position and 0's elsewhere, and $\underline{\mathbf{1}}$ denotes a column vector of 1's of appropriate size, and the two alternative forms are due to the property $\mathbf{Q}\underline{\mathbf{1}} = \underline{\mathbf{0}}$ of the generator of the Markov chain, which gives

$$\begin{aligned} \mathbf{Q}^* \underline{\mathbf{1}} + \mathbf{V} \underline{\mathbf{1}} &= \underline{\mathbf{0}}, \\ \mathbf{Q}^* \underline{\mathbf{1}} &= -\mathbf{V} \underline{\mathbf{1}}, \end{aligned} \quad (43)$$

where $\underline{\mathbf{0}}$ denotes a column vector of 0's of appropriate size.

The expression for $F_i(t)$ follows analytically as

$$\begin{aligned} F_i(t) &= \int_0^t f_i(u) du \\ &= \int_0^t \underline{\mathbf{e_i}} e^{\mathbf{Q}^* u} \mathbf{V} \underline{\mathbf{1}} du \\ &= \left[\underline{\mathbf{e_i}} e^{\mathbf{Q}^* u} \left(\mathbf{Q}^* \right)^{-1} \mathbf{V} \underline{\mathbf{1}} \right]_0^t \\ &= \underline{\mathbf{e_i}} \left(e^{\mathbf{Q}^* t} - \mathbf{I} \right) \left(\mathbf{Q}^* \right)^{-1} \mathbf{V} \underline{\mathbf{1}} \\ &= -\underline{\mathbf{e_i}} \left(e^{\mathbf{Q}^* t} - \mathbf{I} \right) \underline{\mathbf{1}} \\ &= 1 - \underline{\mathbf{e_i}} e^{\mathbf{Q}^* t} \underline{\mathbf{1}}. \end{aligned} \quad (44)$$

In particular, the probability of not being absorbed into $\{S, P\}$ by time t, given start in i, is given by

$$1 - F_i(t) = \underline{\mathbf{e_i}} e^{\mathbf{Q}^* t} \underline{\mathbf{1}}. \quad (45)$$

This result is unsurprising, as we interpret $\underline{\mathbf{u}}e^{\mathbf{Q}^* t}$ as the probability distribution at time t of the Markov chain given initial distribution $\underline{\mathbf{u}}$ at time 0.

Summarizing the above analysis, the hazard rates $\lambda_i(t)$ are given by

$$\lambda_i(t) = \frac{-\underline{\mathbf{e_i}}e^{\mathbf{Q}^* t}\mathbf{Q}^*\underline{\mathbf{1}}}{\underline{\mathbf{e_i}}e^{\mathbf{Q}^* t}\underline{\mathbf{1}}}. \tag{5}$$

B.2 Cause-specific hazard rates (absorption into S or P)

As discussed in Section 2, when an absorption into $\{S, P\}$ occurs, the process transitions to either S or P. We thus define the pseudogenization ($j = P$) and subfunctionalization ($j = S$) cause-specific hazard rates by

$$\lambda_{ij}(t) = \lim_{h \to 0^+} \frac{P(t < T_{\{S,P\}} < t + h, X(T_{\{S,P\}}) = j | T_{\{S,P\}} > t, X(0) = i)}{h} = \frac{f_{ij}(t)}{1 - F_i(t)}, \tag{6}$$

where $f_{ij}(t)$ is the probability density of absorption occurring at time t *and* the absorption occurring into the specific state j, given start in state i. $F_i(t)$ is the corresponding cumulative distribution function. We note that

$$\begin{aligned} f_i(t) &= \sum_{j \in \{S,P\}} f_{ij}(t), \\ \lambda_i(t) &= \sum_{j \in \{S,P\}} \lambda_{ij}(t). \end{aligned} \tag{46}$$

By the analysis of the Markov chain, we have

$$f_{ij}(t) = \left[\underline{\mathbf{e_i}}e^{\mathbf{Q}^* t}\mathbf{V}\right]_j, \tag{47}$$

and so the hazard rates $\lambda_{ij}(t)$ are given by

$$\lambda_{ij}(t) = \frac{\left[\underline{\mathbf{e_i}}e^{\mathbf{Q}^* t}\mathbf{V}\right]_j}{\underline{\mathbf{e_i}}e^{\mathbf{Q}^* t}\underline{\mathbf{1}}}. \tag{7}$$

Further, by carefully applying the law of total probability together with the memoryless property of the Markov chain, it follows (a proof of a more general case is provided in Appendix F) that

$$\lim_{t \to \infty} \lambda_{ij}(t) = \begin{cases} ur + u_c & \text{for } j = P \\ ur & \text{for } j = S. \end{cases} \tag{48}$$

So the cause-specific hazard rates $\lambda_{iS}(t)$ and $\lambda_{iP}(t)$ converge to u_r and $u_r + u_c$ respectively, as $t \to \infty$.

B.3 Average cause-specific hazard rates

As the number of regulatory regions z may be unknown, we now introduce the *average cause-specific hazard rates* that depend on some distribution of z.

First, we define a random variable Z taking integer values from Z_{min} to Z_{max} (possibly 1 and ∞), and let $p_z = P(Z = z)$ be the probability of having z regulatory regions in the duplicate pair of genes. Also, we let $\underline{\mathbf{p}} = [p_z]_{z=Z_{min},\ldots,Z_{max}}$ be the (row) vector recording these probabilities. If data is available, the probabilities p_z can be estimated using

$$p_z = \frac{x_z}{\sum_{z=Z_{min}}^{Z_{max}} x_z} \ , \tag{49}$$

where x_z is the observed number of genes with z regulatory regions [8]. For now, we make no assumptions about the shape of this distribution.

Further, since the rates $\lambda_{ij}(t)$ depend on z, we denote by $\lambda_{ij}^z(t)$ the hazard rates derived for the specific values of z. Note that in the analysis of the subfunctionalization model, we are only interested in the case where we start from state $i = 0$, and so for notational convenience, we let $\lambda_j^z = \lambda_{0j}^z$.

With this notation, for $j \in \{S, P\}$, we define the average hazard rates as

$$\Lambda_j(t) = \sum_{z=Z_{min}}^{Z_{max}} p_z \lambda_j^z(t). \tag{50}$$

B.4 Other measures of interest

We introduce the following notation for other measures of interest. Given that the process starts in state i, we define

- $p_{i,S}$ - the probability of absorption into S,
- $p_{i,P}$ - the probability of absorption into P,
- $m_i = m_i^{(1)}$ - the mean time till absorption into $\{S, P\}$,
- $m_i^{(k)}$ - the k-th moment of the time till absorption into $\{S, P\}$, $k \geq 1$,
- var_i - variance of the time till absorption into $\{S, P\}$.

We then have

$$
\begin{aligned}
p_{i,S} &= \int_{t=0}^{\infty} \underline{\mathbf{e_i}} e^{\mathbf{Q}^* t} \underline{\mathbf{v}}_S dt \\
&= -\underline{\mathbf{e_i}} (\mathbf{Q}^*)^{-1} \underline{\mathbf{v}}_S, && (51) \\
p_{i,P} &= -\underline{\mathbf{e_i}} (\mathbf{Q}^*)^{-1} \underline{\mathbf{v}}_P, && (52) \\
m_i &= \int_{t=0}^{\infty} t \underline{\mathbf{e_i}} e^{\mathbf{Q}^* t} \mathbf{V} \underline{\mathbf{1}} dt \\
&= -\underline{\mathbf{e_i}} (\mathbf{Q}^*)^{-1} \underline{\mathbf{1}}, && (53) \\
m_i^{(k)} &= \int_{t=0}^{\infty} t^k \underline{\mathbf{e_i}} e^{\mathbf{Q}^* t} \mathbf{V} \underline{\mathbf{1}} dt \\
&= (-1)^k k! \underline{\mathbf{e_i}} (\mathbf{Q}^*)^{-k} \underline{\mathbf{1}}, && (54) \\
var_i &= m_i^{(2)} - (m_i)^2, && (55)
\end{aligned}
$$

where (53)–(54) follow by integration by parts and mathematical induction. Note that we have

$$
\begin{aligned}
p_{0,S} &= \sum_{i=1}^{z} S_i^{*z}, && (56) \\
p_{0,P} &= \sum_{i=1}^{z} P_i^{*z}, && (57)
\end{aligned}
$$

where S_i^{*z} and P_i^{*z} are the quantities derived in Section A.

Further, given start in i, define

- $m_{i,S} = m_{i,S}^{(1)}$ - the mean time till absorption into S *given* absorption into S occurs,
- $m_{i,S}^{(k)}$ - the k-th moment of the time till absorption into S *given* absorption into S occurs, $k \geq 1$,
- $m_{i,P} = m_{i,P}^{(1)}$ - the mean time till absorption into P *given* absorption into P occurs,
- $m_{i,P}^{(k)}$ - the k-th moment of the time till absorption into P *given* absorption into P occurs, $k \geq 1$.
- $var_{i,S}$ - variance of the time till absorption into S *given* absorption into S occurs,
- $var_{i,P}$ - variance of the time till absorption into P *given* absorption into P occurs.

We then have, with $p_{i,S}$ and $p_{i,P}$ as derived above, by integration by parts and mathematical induction,

$$\begin{aligned} m_{i,S} &= \int_{t=0}^{\infty} t\underline{\mathbf{e_i}} e^{\mathbf{Q}^* t} \underline{\mathbf{v}}_s dt / p_{i,S} \\ &= \underline{\mathbf{e_i}} (\mathbf{Q}^*)^{(-2)} \underline{\mathbf{v}}_s / p_{i,S}, & (58) \\ m_{i,S}^{(k)} &= \int_{t=0}^{\infty} t^k \underline{\mathbf{e_i}} e^{\mathbf{Q}^* t} \underline{\mathbf{v}}_s dt / p_{i,S} \\ &= (-1)^{(k+1)} k! \underline{\mathbf{e_i}} (\mathbf{Q}^*)^{(-(k+1))} \underline{\mathbf{v}}_s / p_{i,S}, & (59) \\ m_{i,P} &= \underline{\mathbf{e_i}} (\mathbf{Q}^*)^{(-2)} \underline{\mathbf{v}}_P / p_{i,P}, & (60) \\ m_{i,P}^{(k)} &= (-1)^{(k+1)} k! \underline{\mathbf{e_i}} (\mathbf{Q}^*)^{(-(k+1))} \underline{\mathbf{v}}_P / p_{i,P}, & (61) \\ var_{i,S} &= m_{i,S}^{(2)} - (m_{i,S})^2, & (62) \\ var_{i,P} &= m_{i,P}^{(2)} - (m_{i,P})^2. & (63) \end{aligned}$$

C Pseudogenization Rate

Consider the pseudogenization rate, defined in Equation (13) of the main document as,

$$h_P^z(t) = \lim_{h \to 0^+} \frac{P(t < T_P < t + h | T_P > t, X(0) = 0)}{h} = \frac{\tilde{f}(t)}{1 - \tilde{F}(t)},$$

The expression for $\tilde{F}_{ij}(t)$ follows analytically as

$$\begin{aligned} F_{ij}(t) &= \int_0^t \tilde{f}_{ij}(u) du \\ &= \int_0^t \underline{\mathbf{e_i}} e^{\mathbf{Q}^* u} \mathbf{V} \underline{\mathbf{1}} du \\ &= \left[\underline{\mathbf{e_i}} e^{\mathbf{Q}^* u} (\mathbf{Q}^*)^{-1} \mathbf{V} \underline{\mathbf{1}} \right]_0^t \\ &= \underline{\mathbf{e_i}} \left(e^{\mathbf{Q}^* t} - \mathbf{I} \right) (\mathbf{Q}^*)^{-1} \mathbf{V} \underline{\mathbf{1}} \\ &= -\underline{\mathbf{e_i}} \left(e^{\mathbf{Q}^* t} - \mathbf{I} \right) \underline{\mathbf{1}} \\ &= 1 - \underline{\mathbf{e_i}} e^{\mathbf{Q}^* t} \underline{\mathbf{1}}. & (64) \end{aligned}$$

Therefore,

$$\begin{aligned} h_P^z(t) &= \frac{\underline{\mathbf{e}}_0 e^{\mathbf{Q}^* t} \underline{\mathbf{v}}_P}{1 - \int_{u=0}^t \underline{\mathbf{e}}_0 e^{\mathbf{Q}^* u} \underline{\mathbf{v}}_P du} \\ &= \frac{\underline{\mathbf{e}}_0 e^{\mathbf{Q}^* t} \underline{\mathbf{v}}_P}{1 - \underline{\mathbf{e}}_0 \left(e^{\mathbf{Q}^* t} - \mathbf{I} \right) (\mathbf{Q}^*)^{-1} \mathbf{v}_P}. & (65) \end{aligned}$$

Since $\lim_{t\to\infty} e^{\mathbf{Q}^* t} = 0$, the corresponding limits as $t \to \infty$ are

$$\begin{aligned}
\lim_{t\to\infty} h_P^z(t) &= \frac{\lim_{t\to\infty} \underline{\mathbf{e}}_0 e^{\mathbf{Q}^* t} \underline{\mathbf{v}}_P}{1 - \lim_{t\to\infty} \underline{\mathbf{e}}_0 \left(e^{\mathbf{Q}^* t} - \mathbf{I}\right) (\mathbf{Q}^*)^{-1} \mathbf{v}_P} \\
&= \frac{0}{1 + \underline{\mathbf{e}}_0 (\mathbf{Q}^*)^{-1} \mathbf{v}_P} \\
&= 0, \qquad (66)
\end{aligned}$$

and so the rates $h_P^z(t)$ converge to 0 as $t \to \infty$.

Further, the average pseudogenization rate (for randomly distributed z) was defined in equation (16 as

$$H_P(t) = \sum_{z=Z_{min}}^{Z_{max}} p_z h_P^z(t).$$

Notice that for any distribution $\underline{\mathbf{p}} = [p_z]_{z=Z_{min},\ldots,Z_{max}}$ we have

$$\begin{aligned}
\lim_{t\to\infty} H_P(t) &= \lim_{t\to\infty} \sum_{z=Z_{min}}^{Z_{max}} p_z h_P^z(t) \\
&= \sum_{z=Z_{min}}^{Z_{max}} p_z \lim_{t\to\infty} h_P^z(t), \\
&= 0, \qquad (67)
\end{aligned}$$

where the final line follows from the application of equation (66).

D Subfunctionalization survival function and Poisson duplication

In order to fit the model to genome data, we will make use of the survival function implied by our model together with some assumptions about the underlying gene duplication process.

By equation (65), the survival function corresponding to the random variable T_P is given by

$$P(T_P > t) = 1 - \tilde{F}(t) = 1 - \underline{\mathbf{e}}_0 \left(e^{\mathbf{Q}^* t} - \mathbf{I}\right) (\mathbf{Q}^*)^{-1} \mathbf{v}_P. \qquad (68)$$

Note that the data (handled by Hughes and Liberles [8]) contains the counts of the number of surviving duplicates at the current time. However, this number depends on the gene duplication process, which needs to be considered in the analysis. Therefore, to model the gene duplication process, we introduce a random variable N and assume that N follows a Poisson distribution with some parameter β_0. The Poisson rate β_0 is an additional parameter that we introduce here in order to fit our model to the data.

Specifically, we assume that N counts the number of duplication events that occur in a time interval of length $0.01s$ - s being the expected number of substitutions per silent site. Also, we let $Y(t)$ be a random variable tracking the number of duplicates which have survived to the current time, having been duplicated at time t in the past. Then, we have

$$\begin{aligned}
P(Y(t) = y) &= \sum_{n \geq y} P(Y(t) = y | N = n) P(N = n) \\
&= \sum_{n \geq y} (1 - \tilde{F}(t))^y \tilde{F}(t)^{n-y} \binom{n}{y} P(N = n) \\
&= (1 - \tilde{F}(t))^y \sum_{n \geq y} \tilde{F}(t)^{n-y} \left(\frac{n!}{y!(n-y)!} \right) \frac{\beta_0^n}{n!} e^{-\beta_0} \\
&= \frac{(1 - \tilde{F}(t))^y e^{-\beta_0}}{y!} \sum_{n \geq y} \frac{\tilde{F}(t)^{n-y} \beta_0^n}{(n-y)!} \\
&= \frac{(1 - \tilde{F}(t))^y e^{-\beta_0}}{y!} \beta_0^y \sum_{n \geq 0} \frac{(\tilde{F}(t)\beta_0)^n}{n!} \\
&= \frac{(1 - \tilde{F}(t))^y e^{-\beta_0}}{y!} \beta_0^y e^{\tilde{F}(t)\beta_0} \\
&= \frac{((1 - \tilde{F}(t))\beta_0)^y}{y!} e^{-\beta_0(1 - \tilde{F}(t))}, \qquad (69)
\end{aligned}$$

where $\tilde{F}(t)$ is defined in (68).

Note that (69) defines a Poisson random variable with parameter

$$\beta(t) = \beta_0(1 - F(t)). \qquad (70)$$

With this result, calculating the likelihood of the data given the parameters is straight forward, and computationally tractable, as we only need to multiply over the probabilities for each of the data bins.

Next, in order to fit the parameters β_0, u_r, u_c and z to the data set, we use the maximum likelihood method with the log likelihood given by

$$log(L_\theta) = \sum_i D_i \log(\beta(s_i) - \beta(s_i) - \Gamma \log(D_i + 1), \qquad (71)$$

where D_i is the count in the ith bin of the data set, and s_i is the associated cumulative number of silent substitutions per silent site, used as a proxy for time.

E Comparison of pseudogenization rate to existing phenomenological approximations

Here, we compare the qualitative and quantitative features of the pseudogenization rate (13) derived here to the phenomenological approximations of Konrad et al [12] and Teufel et al [11]. We analyze the parameter space of each of the phenomenological rate functions to find correspondences between the phenomenological and mechanistic parameters. We find that both of the approximations have a good qualitative correspondance to the pseuodgenization rate (13) derived here, but the approximation in Teufel et al [11] is in particularly good agreement with our rate.

We will be making use of the continuation of h taking the domain from $\mathbb{R}^+ \cup \{0\}$ to $\mathbb{R}$ (the expession for the function remains the same), and making use of the following result, which we prove a more general form of later in Appendix G

$$\lim_{t\to-\infty} h_P^z(t) = 2(u_c + zu_r). \tag{72}$$

We will also use the result of equation (66), which was proved in the main body of the work and is restated here for convenience

$$\lim_{t\to\infty} h_P^z(t) = 0.$$

E.1 Konrad et al [12] approximation

First, consider the approximation in Konrad et al [12], which we denote $h_K(t)$, given by

$$h(t) \approx h_K(t) = fe^{-bt^c} + d. \tag{73}$$

The first and second derivate of $h_K(t)$ are given by

$$h'_K(t) = -fbct^{c-1}e^{-bt^c} \tag{74}$$

$$\begin{aligned} h''_K(t) &= f\left(b^2c^2t^{2(c-1)}e^{-bt^c} - b(c-1)ct^{c-2}e^{-bt^c}\right) \\ &= bct^{c-2}e^{-bt^c}\left(c(bt^c-1)+1\right). \end{aligned} \tag{75}$$

Below, we show that the pseudogenization rate $h_P^z(t)$ defined in (13) can be approximated using a function of the form (73). We find sets of parameters f, b, c and d that correspond well to the qualitative behaviour of h_P^z in each of the three cases $\gamma < \gamma_{\text{crit}}$, $\gamma = \gamma_{\text{crit}}$ and $\gamma > \gamma_{\text{crit}}$.

E.1.1 $d = 0$

Firstly, we note that since $\lim_{t\to\infty} e^{-t} = 0$, then $\lim_{t\to\infty} h_K(t) = d$. The corresponding limit for the pseudogenization rate is $\lim_{t\to\infty} h_P(t) = 0$, and so we require $d = 0$ if we wish to match the long-run behaviour of the approximation $h_K(t)$ to that of $h_P(t)$.

E.1.2 $c = 1$ corresponds well to $\gamma \geq \gamma_{\text{crit}}$

Next, we note that the parameter c of $h_K(t)$ is a shape parameter, and when $c = 1$, $h_K(t)$ is exponential. Recalling Examples 5.2 and 5.3, we note that $h_P(t)$ behaves like an exponential when $\gamma \geq \gamma_{\text{crit}}$, so we have good qualitative correspondance between the $h_K(t)$ when $c = 1$ and $h_P(t)$ when $\gamma \geq \gamma_{\text{crit}}$.

Figure 1 shows a plot of the $h_K(t)$ with $c = 1$.

E.1.3 $c > 1$ corresponds reasonably well to $\gamma < \gamma_{\text{crit}}$

When $c > 1$, $h'_K(t)$ given in equation (74) has a single root at $t = 0$, while $h''_K(t)$, given in equation (75) has two roots, one at $t = 0$ and one when $bt^c - 1 = 1/c$, which occurs for some $t > 0$. This means that there is a change of concavity at some $t > 0$, before a flattening out as $t \to 0^+$. This is qualitatively similar to $h^z_P(t)$ for $\gamma < \gamma_{\text{crit}}$.

However, for any realistic parameter set $h^z_P(t)$ is sigmoidally shaped, with $h^{z\prime}_P(t) \neq 0$ for all t. As a result, the flattening out behaviour around $t = 0$ is never quite matched by the pseudogenization rate function.

Figure 2 shows $h_K(t)$ for $c = 2 > 1$.

E.1.4 $0 < c < 1$ corresponds somewhat well to $\gamma > \gamma_{\text{crit}}$

When $c < 1$ the derivative $h'_K(t) \to -\infty$ as $t \to 0^+$. This gives an exponential-like function, which is becoming vertical as $t \to 0^+$. This is qualitatively most similar to the function $h^z_P(t)$ when $\gamma > \gamma_{\text{crit}}$. However, $h_K(t)$ gives a faster decline in the rate for small t.

Figure 3 shows $h_K(t)$ for $0 < c = 0.5 < 1$.

The remaining cases ($c \leq 0$) are not of interest here, since $c = 0$ has no dependence on time, and $c < 0$ would give a rate function which increased in t. Neither of these behaviours is suitable for modeling pseudogenization rate under subfunctionalization.

E.2 Teufel et al [11] approximation

We now consider the approximation in Teufel et al [11] which we denote $h_T(t)$, given by

$$h(t) \approx h_T(t) = u + \frac{be^{a-t}}{1 + e^{a-t}}. \tag{76}$$

The first and second derivates of $h_T(t)$ are given by

$$h'_T(t) = -\frac{be^{a+t}}{(e^a + e^t)^2} \tag{77}$$

$$h''_T(t) = \frac{be^{a+t}(e^t - e^a)}{(e^a + e^t)^3}. \tag{78}$$

E.2.1 Comparison of overall shape of $h_T(t)$ to that of $h_P^z(t)$ for different parameter sets

First we examine the overall shape of $h_T(t)$, and compare it to that of $h_P^z(t)$. Note that the first derivative $h'_T(t)$ has no roots outside of the trivial case $b = 0$. The second derivative has a root at $t = a$, and hence this is a sigmoidal function. Since $h_P^z(t)$ has a sigmoidal shape for most parameter sets, we can expect to find a good qualitative agreement between the two functions.

Since the second derivative $h''_T(t)$ defined in equation (78) has a root at $t = a$, selecting $a < 0$ results in a change in concavity for $h_T(t)$ in non-physical time. This is equivalent to the behaviour of $h_P^z(t)$ in the case $\gamma > \gamma_{\text{crit}}$.

Similarly, when $a = 0$ the change in concavity for $h_T(t)$ occurs at $t = 0$. This is equivalent to the behaviour of $h_P^z(t)$ in the case $\gamma = \gamma_{\text{crit}}$.

When $a > 0$ there is a change in the concavity of $h_T(t)$ at $t = a > 0$, and this is equivalent to the behaviour of $h_P^z(t)$ when $\gamma < \gamma_{\text{crit}}$.

Teufel et al [11] suggest that parameter sets with $a > 1$ (and $b > 0$) correspond to subfunctionalization, while those with $a < 1$ (and $b > 0$) correspond to neofunctionalization. Our analysis shows that both of these parameter sets should correspond well to subfunctionalization, and this suggests that distinguishing between sub- and neofunctionalization in a modelling framework will present a major challenge.

Below, we perform a more detailed analysis, allowing us to choose parameters which match specific features of the two functions – the initial values, the limits as $t \to \pm\infty$, and the location of the point of inflection. This allows us to easily choose parameters for $h_T(t)$ defined in (76 that exhibit similar behaviour to our pseudogenization rate $h_P^z(t)$ defined in (13).

E.2.2 Matching the limit as $t \to \infty$ of $h_T(t)$ to that of $h_P^z(t)$

Similarly to the approximation in Konrad et al [12], this function has a constant term, u which is equal to its limit as $t \to \infty$. Thus we can choose

$$u = \lim_{t \to \infty} h_P^z(t) = 0 \tag{79}$$

in order to match the long term behaviour of $h_T(t)$ to that of $h_P^z(t)$ for any parameter set.

E.2.3 Matching the location of the point of inflection of $h_T(t)$ to that of $h_P^z(t)$

Recall from Section E.2.1 that the point of inflection of $h_T(t)$ occurs at the point $t = a$. While we do not have an analytic solution for the point of inflection of $h_P^z(t)$, we know that it occurs when $h_P^{z\prime\prime}(t) = 0$, and we can solve for t numerically. Thus the parameter a can be chosen such that

$$h_P^{z\prime\prime}(a) = 0. \tag{80}$$

This results in the point of inflection for $h_T(t)$ and $h_P^z(t)$ occuring at the same value $t = a$.

E.2.4 Matching the initial value of $h_T(t)$ to that of $h_P^z(t)$

The initial value of $h_T(t)$ is (assuming $u = 0$)

$$h_T(0) = \frac{be^a}{1 + e^a}.$$

Since $h_P^z(t) = 2u_c$, we set $h_T(0) = h_P^z(0)$ by choosing b according to

$$\frac{be^a}{1 + e^a} = 2u_c$$
$$\text{i.e. } b = 2\left(\frac{u_c}{e^a} + 1\right). \tag{81}$$

E.2.5 Matching the limit as $t \to -\infty$ of $h_T(t)$ to that of $h_P^z(t)$

An alternative choice for the parameter b makes use of our result (72). This allows the parameter b to be selected independent of a, provides some additional flexibility in choosing parameter sets to match the pseudogenization rate (13). First we note

$$\lim_{t \to -\infty} h_T(t) = u + b,$$

on the other hand, recalling the result (72)

$$\lim_{t \to -\infty} h_P^z = 2(u_c + zu_r).$$

Thus (assuming $u = 0$) we can choose

$$b = 2(u_c + zu_r) \tag{82}$$

to match the limits as $t \to -\infty$. In this case, we are free to choose a according to either equation (80) or (81).

Although the behaviour of the function in negative t has no physical relevence, for sigmoidal functions the limit as $t \to -\infty$ is generally more descriptive than the value of the function at some fixed t. The limits at $\pm\infty$ together with the point of inflection describe all of the main features of the function besides its steepness about the point of inflection.

E.2.6 Choosing parameters to fit $h_T(t)$ to $h_P(t)$

We have derived four equations which can be used to match the behaviour of the approximation in Teufel et al [11] defined in (76) to that of the pseudogenization rate function (13) defined here.

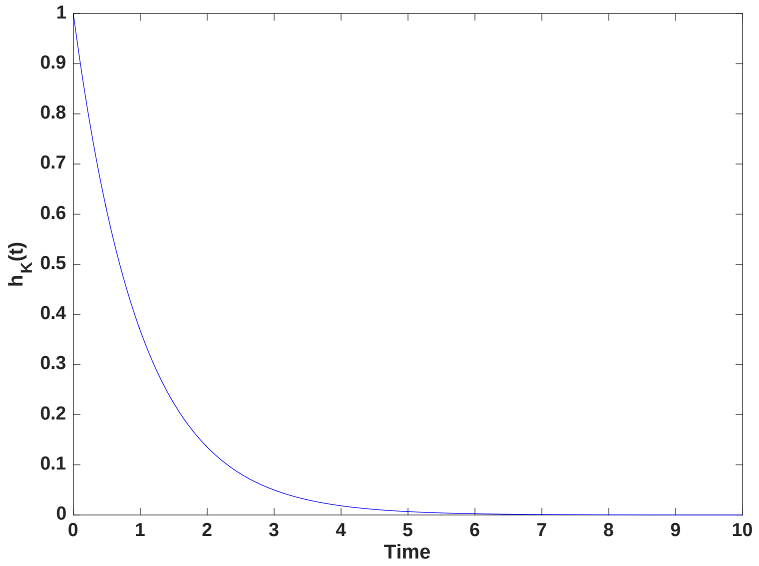

Figure 1: The approximation in Konrad et al [12] with shape parameter $c = 1$. The remaining parameters were $f = b = 1$, $d = 0$

It is usually only possible to satisfy three of the four equations, and it is assumed in the derivation of equations (82) and (81) that $u = 0$ (i.e. that equation (79) is satisfied). We are left to choose which two of the remaining three equations, (82, 81, 80) to to satisfy. The choice can be made by considering which qualitative features of $h_P^z(t)$ we want $h_T(t)$ to preserve,

- to preserve the initial value (value at $t = 0$), we must satisfy equation (81),
- to preserve the value of t at which the point of inflection occurs, we must satisfy equation (80),
- and to preserve the limit as $t \to -\infty$ we must satisfy (82).

Taking combinations of these we can get three different parameter sets that will lead to good qualitative agreement between $h_T(t)$ and $h_P^z(t)$.

E.3 Summary

The approximation in Konrad et al [12], denoted $h_K(t)$ and defined in equation (73) is qualitatively quite similar to our pseudogenization rate function $h_P^z(t)$, defined in equation (13). However, there are some differences between the behaviour of the $h_K(t)$ and $h_P^z(t)$ for small t. The best agreement is achieved

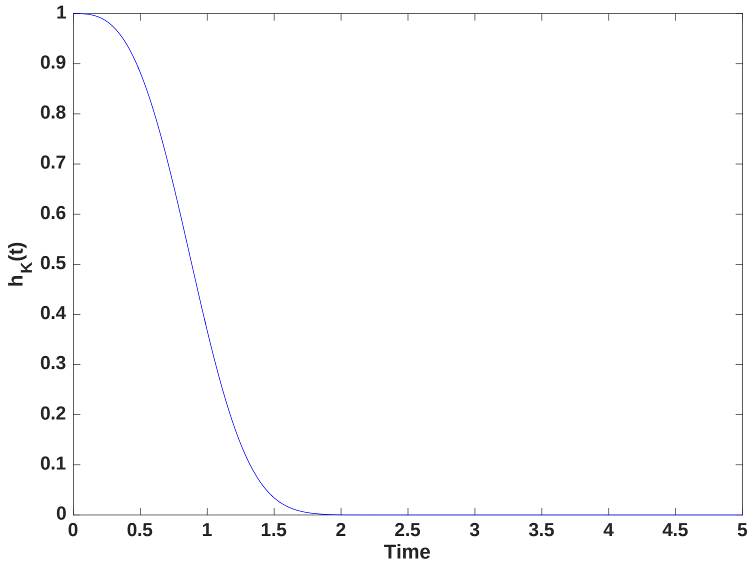

Figure 2: The approximation in Konrad et al [12] with shape parameter $c = 2$. The remaining parameters were $f = b = 1$, $d = 0$

when $c = 1$ and $h_K(t)$ reduces to an exponential, which is the arguably the least interesting behaviour available to the pseudogenization rate function $h^z_P(t)$.

The approximation in Teufel et al [11], denoted $h_K(t)$ and defined in equation (76) is more flexible than $h_K(t)$, and is qualitatively very similar to $h^z_P(t)$. Like $h^z_P(t)$, $h_K(t)$ can have a point of inflection at any value of t

F Proof of equation (48)

First, we prove the following more general result, of which equation (48) is a corollary.

Lemma 1. *Let $X(t)$ be an absorbing CTMC with some finite state space $\mathcal{S} = \{1, ..., m\} \cup \mathcal{A}$, with $\mathcal{A}$ being the set of absorbing states, and generator $\mathbf{Q} = [q_{ij}]_{i,j \in S}$ such that $q_{ij} = 0$ for all $i, j \in \{1, ..., m\}$, and state m being accessible from any $i \in \{1, ..., m-1\}$*

Let $T_i = \inf\{t > 0 : X(t) = i\}$ be the first time state i is visited, $T_{\mathcal{A}} = \inf\{t > 0 : X(t) \in \mathcal{A}\}$ be the first time the process gets absorbed into some state in $\mathcal{A}$, and consider cause-specific hazard rates given by

$$\lambda_{ij}(t) = \lim_{h \to 0^+} \frac{P(t < T_{\mathcal{A}} < t + h, X(T_{\mathcal{A}}) = j | T_{\mathcal{A}} > t, X(0) = i)}{h} = \frac{f_{ij}(t)}{1 - F_i(t)} \ .$$

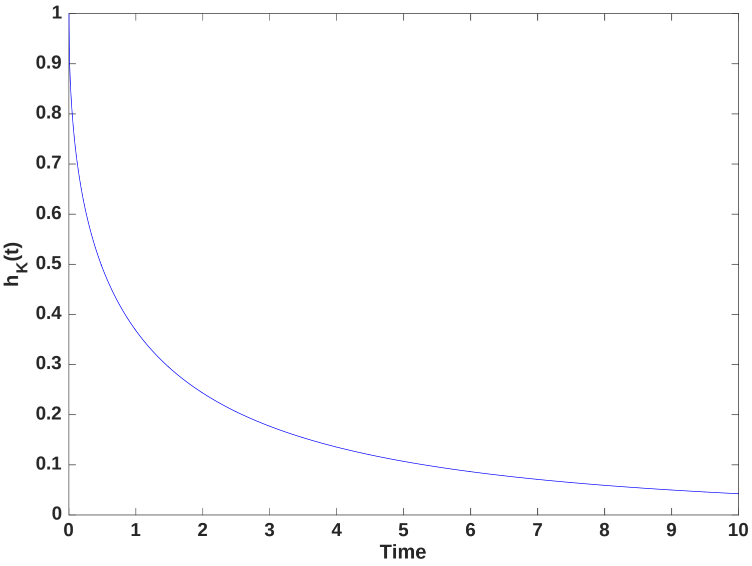

Figure 3: The approximation in Konrad et al [12] with shape parameter $c = 2$. The remaining parameters were $f = b = 1$, $d = 0$

Then, for any $i \in \{1, ..., m\}, j \in \mathcal{A}$

$$\lim_{t\to\infty} \lambda_{ij}(t) = q_{mj}.$$

Proof. Denote the events $A_t^h = \{t < T_{\mathcal{A}} < t+h\}$, $B_t = \{T_{\mathcal{A}} > t\}$, $C_t = \{T_m \leq t\}$, $\overline{C}_t = \{T_m > t\}$. By the law of total probability and the memoryless property of the Markov chain, and since

$$\lim_{t\to\infty} P(C_t|B_t, X(0) = i) = 1 \quad \text{and} \quad \lim_{t\to\infty} P(\overline{C}_t|B_t, X(0) = i) = 0, \tag{83}$$

it follows that the limits of $\lambda_{ij}(t)$ as $t \to \infty$ are

$$\begin{aligned}
\lim_{t\to\infty} \lambda_{ij}(t) &= \lim_{t\to\infty} \lim_{h\to 0^+} \frac{P(A_t^h, X(T_{\mathcal{A}}) = j | B_t, X(0) = i)}{h} \\
&= \lim_{t\to\infty} \lim_{h\to 0^+} \frac{P(A_t^h, X(T_{\mathcal{A}}) = j | B_t, X(0) = i, C_t) P(C_t | B_t, X(0) = i)}{h} \\
&\quad + \lim_{t\to\infty} \lim_{h\to 0^+} \frac{P(A_t^h, X(T_{\mathcal{A}}) = j | B_t, X(0) = i, \overline{C}_t) P(\overline{C}_t | B_t, X(0) = i)}{h} \\
&= \lim_{t\to\infty} \left[P(C_t | B_t, X(0) = i) \lim_{h\to 0^+} \frac{P(A_t^h, X(T_{\mathcal{A}}) = j | B_t, X(0) = i, C_t)}{h} \right] \\
&\quad + \lim_{t\to\infty} \left[P(\overline{C}_t | B_t, X(0) = i) \lim_{h\to 0^+} \frac{P(A_t^h, X(T_{\mathcal{A}}) = j | B_t, X(0) = i, \overline{C}_t)}{h} \right] \\
&= \lim_{t\to\infty} \lim_{h\to 0^+} \frac{P(A_t^h, X(T_{\mathcal{A}}) = j | B_t, X(0) = i, C_t)}{h} \\
&= \lim_{t\to\infty} \lim_{h\to 0^+} \frac{P(t < T_{\mathcal{A}} < t+h, X(T_{\mathcal{A}}) = j | (T_{\mathcal{A}} > t, X(0) = i, T_m \le t)}{h} \\
&= \lim_{t\to\infty} \lim_{h\to 0^+} \frac{P(t < T_{\mathcal{A}} < t+h, X(T_{\mathcal{A}}) = j | X(t) = m, X(0) = i)}{h} \\
&= \lim_{t\to\infty} \lim_{h\to 0^+} \frac{P(t < T_{\mathcal{A}} < t+h, X(T_{\mathcal{A}}) = j | X(t) = m)}{h}
\end{aligned}$$

$$\begin{aligned}
&= \lim_{t\to\infty} \lim_{h\to 0^+} \frac{P(X(t+h) = j | X(t) = m)}{h} \\
&= \lim_{h\to 0^+} \frac{P(X(h) = j | X(0) = m)}{h} \\
&= q_{m,j}
\end{aligned}$$

□

Corollary 1. *Consider the CTMC defined in Section 1, by the application of Lemma 1 we have the result*

$$\lim_{t\to\infty} \lambda_{ij}(t) = \begin{cases} ur + u_c & \text{for } j = P \\ ur & \text{for } j = S. \end{cases}$$

G Proof of equation (72)

Again, we prove the more general result, of which equation (72) is a corollary.

Lemma 2. *Let $X(t)$ be an absorbing CTMC with some finite state space $\mathcal{S} = \{1, ..., n\} \cup \mathcal{A}$, with $\mathcal{A}$ being the set of absorbing states, initial distribution $\underline{\alpha} = [\alpha_i]_i$, and some generator $\mathbf{Q} = [q_{ij}]_{i,j\in S}$ such that*

$$\mathbf{Q} = \left[\begin{array}{c|c} \mathbf{Q}^* & \mathbf{V} \\ \hline \mathbf{O} & \mathbf{O} \end{array} \right],$$

with $\mathbf{Q}^* = [q_{ij}]_{i,j\in\{1,\ldots,n\}}$, $\mathbf{V} = [q_{ij}]_{i\in\{1,\ldots,n\},j\in\mathcal{A}} = [\underline{\mathbf{v}}_j]_{j\in\mathcal{A}}$.

For any $k \in \mathcal{A}$*, define*

$$h_k(t) = \frac{\underline{\alpha} e^{\mathbf{Q}^* t}\underline{\mathbf{v}}_k}{1 - \int_{u=0}^{t} \underline{\alpha} e^{\mathbf{Q}^* u}\underline{\mathbf{v}}_k du} \text{ for all } t \in \mathbb{R}. \tag{84}$$

For $t \geq 0$*,* $h_k(t)$ *is interpreted as the instantaneous rate of transition into state* k *given that the process has not yet been absorbed into state* k*.*

Then,

$$\lim_{t\to\infty} h_k(t) = 0,$$

and,

$$\lim_{t\to-\infty} h_k(t) = -d_m,$$

where d_m *is the eigenvalue of* $\mathbf{Q}^*$ *with largest absolute value.*

Proof. The result $\lim_{t\to\infty} h_p(t) = 0$ follows by argument analogous to the derivation of equation (66).

The proof for the limit as $t \to -\infty$ is more involved, and we split it into the case where $\mathbf{Q}^*$ is diagonalizable, and the case where it is not.

G.0.1 $\mathbf{Q}^*$ diagonalizable

Since $\mathbf{Q}$ is diagonalizable we let

$$\mathbf{Q}^* = \mathbf{A}^{-1}\mathbf{D}\mathbf{A},$$

where $\mathbf{D}$ is a diagonal matrix of the eigenvalues of $\mathbf{Q}^*$, and denote the ith eigenvector of $\mathbf{Q}^*$ by d_i.

Before making use of the diagonalization, we note that

$$\lim_{t\to-\infty} h_k(t) = \lim_{t\to-\infty} \frac{\underline{\alpha} e^{\mathbf{Q}^* t}\underline{\mathbf{v}}_k}{1 - \underline{\alpha}\left(e^{\mathbf{Q}^* t} - \mathbf{I}\right)(\mathbf{Q}^*)^{(-1)}\underline{\mathbf{v}}_k}$$

is of indeterminent form, and so we apply l'Hôpital's rule to get

$$\lim_{t\to-\infty} h_k(t) = \lim_{t\to-\infty} \frac{\underline{\alpha} e^{\mathbf{Q}^* t}\mathbf{Q}^*\underline{\mathbf{v}}_k}{-\underline{\alpha} e^{\mathbf{Q}^* t}\underline{\mathbf{v}}_k}.$$

Next, making use of the diagonalization, we have

$$\begin{aligned}
\lim_{t\to-\infty} h_k(t) &= \lim_{t\to-\infty} \frac{\underline{\alpha} e^{\mathbf{A}^{-1}\mathbf{D}\mathbf{A}t}\mathbf{A}^{-1}\mathbf{D}\mathbf{A}\underline{\mathbf{v}}_k}{-\underline{\alpha} e^{\mathbf{A}^{-1}\mathbf{D}\mathbf{A}t}\underline{\mathbf{v}}_k} \\
&= \lim_{t\to-\infty} \frac{\underline{\alpha}\mathbf{A}^{-1} e^{\mathbf{D}t}\mathbf{A}\mathbf{A}^{-1}\mathbf{D}\mathbf{A}\underline{\mathbf{v}}_k}{-\underline{\alpha}\mathbf{A}^{-1} e^{\mathbf{D}t}\mathbf{A}\underline{\mathbf{v}}_k} \\
&= \lim_{t\to-\infty} \frac{\underline{\alpha}\mathbf{A}^{-1} e^{\mathbf{D}t}\mathbf{D}\mathbf{A}\underline{\mathbf{v}}_k}{-\underline{\alpha}\mathbf{A}^{-1} e^{\mathbf{D}t}\mathbf{A}\underline{\mathbf{v}}_k} \\
&= \lim_{t\to-\infty} \frac{\sum_j [\underline{\alpha}\mathbf{A}^{-1}]_j e^{d_j t} d_j [\mathbf{A}\underline{\mathbf{v}}_k]_j}{\sum_l -[\underline{\alpha}\mathbf{A}^{-1}]_l e^{d_l t}[\mathbf{A}\underline{\mathbf{v}}_k]_l}.
\end{aligned} \tag{85}$$

Further, denoting the eigenvalue of maximum absolute real value $d_{\rm m}$ we divide the top and bottom of equation (85) by $e^{d_m t}$ to get

$$\lim_{t\to-\infty} h_k(t) = \lim_{t\to-\infty} \frac{\sum_j [\underline{\alpha}\mathbf{A}^{-1}]_j e^{(d_j-d_{\rm m})t} d_j [\mathbf{A}\underline{\mathbf{v}}_k]_j}{-\sum_l [\underline{\alpha}\mathbf{A}^{-1}]_l e^{(d_l-d_{\rm m})t} [\mathbf{A}\underline{\mathbf{v}}_k]_l}. \tag{86}$$

Consider the numerator of equation (86),

$$\begin{aligned}
& \lim_{t\to-\infty} \sum_j [\underline{\alpha}\mathbf{A}^{-1}]_j e^{(d_j-d_{\rm m})t} d_j [\mathbf{A}\underline{\mathbf{v}}_k]_j \\
= \; & [\underline{\alpha}\mathbf{A}^{-1}]_m d_m [\mathbf{A}\underline{\mathbf{v}}_k]_m + \lim_{t\to-\infty} \sum_{j\neq m} [\underline{\alpha}\mathbf{A}^{-1}]_j e^{(d_j-d_{\rm m})t} d_j [\mathbf{A}\underline{\mathbf{v}}_k]_j \\
= \; & d_m [\alpha\mathbf{A}^{-1}]_m [\mathbf{A}\underline{\mathbf{v}}_k]_m, \qquad (87)
\end{aligned}$$

where the final step follows from equation the fact that the eigenvalues of $\mathbf{Q}^*$ are necessarily negative, and that $\lim_{t\to\infty} e^{\mathbf{Q}^* t} = 0$.

Now consider the denominator of equation (86),

$$\begin{aligned}
& -\lim_{t\to-\infty} \sum_l [\underline{\alpha}\mathbf{A}^{-1}]_l e^{(d_l-d_{\rm m})t} [\mathbf{A}\underline{\mathbf{v}}_k]_m \\
= \; & -\left([\underline{\alpha}\mathbf{A}^{-1}]_m [\mathbf{A}\underline{\mathbf{v}}_k]_l + \lim_{t\to-\infty} \sum_{l\neq m} [\underline{\alpha}\mathbf{A}^{-1}]_l e^{(d_l-d_{\rm m})t} [\mathbf{A}\underline{\mathbf{v}}_k]_l \right) \\
= \; & -[\alpha\mathbf{A}^{-1}]_m [\mathbf{A}\underline{\mathbf{v}}_k]_m. \qquad (88)
\end{aligned}$$

Combining equations (87) and (88) we have

$$\begin{aligned}
\lim_{t\to-\infty} h_k(t) &= \frac{d_m [\underline{\alpha}\mathbf{A}^{-1}]_m [\mathbf{A}\underline{\mathbf{v}}_k]_m}{-[\underline{\alpha}\mathbf{A}^{-1}]_m [\mathbf{A}\underline{\mathbf{v}}_k]_m} \\
&= -d_m. \qquad (89)
\end{aligned}$$

G.0.2 $\mathbf{Q}^*$ not diagonalizable

Let

$$\mathbf{Q}^* = \mathbf{P}^{-1}\mathbf{J}\mathbf{P} \tag{90}$$

be the Jordan canonical form of $\mathbf{Q}^*$, with

$$\mathbf{J} = [\mathbf{J}_1 \oplus \ldots \oplus \mathbf{J}_n], \tag{91}$$

where n is the number of unique eigenvalues of $\mathbf{Q}^*$, and each $\mathbf{J}_i$ is a Jordan block. Here $\oplus$ represents the matrix direct sum.

Each block $\mathbf{J}_i$ is associated with a unique eigenvalue of $\mathbf{Q}^*$, denoted d_i. If d_i has algebraic multiplicity a_i, then block $\mathbf{J}_i$ has matrix size $a_i \times a_i$ and $\mathbf{J}_i$ has the form

$$\mathbf{J_i} = \begin{bmatrix} d_i & 1 & & & \\ & d_i & 1 & & \\ & & \ddots & \ddots & \\ & & & d_i & 1 \\ & & & & d_i \end{bmatrix}. \tag{92}$$

By the same arugment as for the diagonalizable case (applying l'Hôpital's rule), we have

$$\lim_{t\to-\infty} h_k(t) = \lim_{t\to-\infty} \frac{\underline{\alpha}\mathbf{P}^{-1}e^{\mathbf{J}t}\mathbf{J}\mathbf{P}\underline{\mathbf{v}}_k}{-\underline{\alpha}\mathbf{P}^{-1}e^{\mathbf{J}t}\mathbf{P}\underline{\mathbf{v}}_k}. \tag{93}$$

We note that,

$$\begin{aligned} e^{\mathbf{J}t} &= [e^{\mathbf{J}_1} \oplus \ldots \oplus e^{\mathbf{J}_n}] \\ &= [e^{d_1 t}\mathbf{K}_1 \oplus \ldots \oplus e^{d_n t}\mathbf{K}_n], \end{aligned} \tag{94}$$

where,

$$\mathbf{K}_i = \begin{bmatrix} 1 & t & \frac{t^2}{2!} & \ldots & \frac{t^{a_i-1}}{(a_i-1)!} \\ & 1 & t & \ldots & \frac{t^{a_i-2}}{(a_i-2)!} \\ & & \ddots & \ddots & \vdots \\ & & & 1 & t \\ & & & & 1 \end{bmatrix}. \tag{95}$$

We can rewrite equation (93) as a sum,

$$\lim_{t\to-\infty} h_k(t) = \lim_{t\to-\infty} \frac{\sum_j [\underline{\alpha}\mathbf{P}^{-1}]_j \sum_i \left[[e^{d_1 t}\mathbf{K}_1 \oplus \ldots \oplus e^{d_n t}\mathbf{K}_n]\mathbf{J}\right]_{ji} [\mathbf{P}\underline{\mathbf{v}}_k]_j}{-\sum_l [\underline{\alpha}\mathbf{P}^{-1}]_l \sum_g [e^{d_1 t}\mathbf{K}_1 \oplus \ldots \oplus e^{d_n t}\mathbf{K}_n]_{lg}[\mathbf{P}\underline{\mathbf{v}}_k]_l}. \tag{96}$$

Next, we divide the top and bottom of equation (96) by $e^{d_m t}$, where d_m is the eigenvalue of $\mathbf{Q}^*$ with maximum absolute value to get

$$\lim_{t\to-\infty} h_k(t) = \lim_{t\to-\infty} \frac{\sum_j [\underline{\alpha}\mathbf{P}^{-1}]_j \sum_i \left[[e^{(d_1-d_m)t}\mathbf{K}_1 \oplus \ldots \oplus K_m \oplus \ldots \oplus e^{(d_n-d_m)t}\mathbf{K}_n]\mathbf{J}\right]_{ji} [\mathbf{P}\underline{\mathbf{v}}_k]_j}{-\sum_l [\underline{\alpha}\mathbf{P}^{-1}]_l \sum_g [e^{(d_1-d_m)t}\mathbf{K}_1 \oplus \ldots \oplus K_m \oplus \ldots \oplus e^{(d_n-d_m)t}\mathbf{K}_n]_{lg}[\mathbf{P}\underline{\mathbf{v}}_k]_l}. \tag{97}$$

Now, we bring the limit inside the (finite) sums, and inside the matrix direct sum. Noting that e^t approachs zero much faster than any of the matrix entries as $t \to -\infty$, we have

$$\lim_{t\to-\infty} h_k(t) = \frac{\sum_j [\underline{\alpha}\mathbf{P}^{-1}]_j \sum_i [[\mathbf{0} \oplus \ldots \oplus \lim_{t\to-\infty} K_m \oplus \ldots \oplus \mathbf{0}]\mathbf{J}]_{ji} [\mathbf{P}\underline{\mathbf{v}}_k]_j}{-\sum_l [\underline{\alpha}\mathbf{P}^{-1}]_l \sum_g [\mathbf{0} \oplus \ldots \oplus \lim_{t\to-\infty} K_m \oplus \ldots \oplus \mathbf{0}]_{lg}[\mathbf{P}\underline{\mathbf{v}}_k]_l}. \tag{98}$$

If we let j_1, j_{a_m} be the first and last index associated with the block $\mathbf{K}_m$ respectively, we can write

$$\lim_{t\to-\infty} h_k(t) = \frac{\sum_{j=j_1}^{j_{a_m}} [\underline{\alpha}\mathbf{P}^{-1}]_j \sum_{i=j_1}^{j-1} [\lim_{t\to-\infty} K_m \mathbf{J}_m]_{ji} [\mathbf{P}\underline{\mathbf{v}}_k]_j}{-\sum_{l=j_1}^{j_{a_m}} [\underline{\alpha}\mathbf{P}^{-1}]_l \sum_{g=j_1}^{l-1} [\lim_{t\to-\infty} K_m]_{lg} [\mathbf{P}\underline{\mathbf{v}}_k]_l}. \tag{99}$$

If $a_m = 1$, then $\mathbf{K}_m = 1$ and $\mathbf{J}_m = d_m$, and equation (99) reduces to $\lim_{t\to-\infty} h_k(t) = d_m$, and the proof is complete. Otherwise, by carefully considering the form of K_m and J_m, we get

$$\lim_{t\to-\infty} h_k(t) = \frac{\sum_{j=j_1}^{j_{a_m}} [\underline{\alpha}\mathbf{P}^{-1}]_j \lim_{t\to-\infty}(d_m + \sum_{i=j_1}^{j-1} \frac{t^{i-1}}{(i-1)!}(1+\frac{td_m}{i}))[\mathbf{P}\underline{\mathbf{v}}_k]_j}{-\sum_{l=j_1}^{j_{a_m}} [\underline{\alpha}\mathbf{P}^{-1}]_l \lim_{t\to-\infty}(1 + \sum_{g=j_1}^{l-1} \frac{t^g}{g!})[\mathbf{P}\underline{\mathbf{v}}_k]_l}, \tag{100}$$

then dividing the top and bottom by t^{a_m-1} we get

$$\lim_{t\to-\infty} h_k(t) = \frac{\sum_{j=j_1}^{j_{a_m}} [\underline{\alpha}\mathbf{P}^{-1}]_j \lim_{t\to-\infty}(d_m t^{1-a_m} + \sum_{i=j_1}^{j-1} \frac{t^{i-a_m}}{(i-1)!}(1+\frac{td_m}{i}))[\mathbf{P}\underline{\mathbf{v}}_k]_j}{-\sum_{l=j_1}^{j_{a_m}} [\underline{\alpha}\mathbf{P}^{-1}]_l \lim_{t\to-\infty}(t^{1-a_m} + \sum_{g=j_1}^{l-1} \frac{t^{g-a_m}}{g!})[\mathbf{P}\underline{\mathbf{v}}_k]_l}. \tag{101}$$

After taking the limit we're left with

$$\begin{aligned}\lim_{t\to-\infty} h_k(t) &= \frac{[\underline{\alpha}\mathbf{P}^{-1}]_{a_m} \frac{d_m}{(a_m-1)!} [\mathbf{P}\underline{\mathbf{v}}_k]_{a_m}}{-[\underline{\alpha}\mathbf{P}^{-1}]_{a_m} \frac{1}{(a_m-1)!} [\mathbf{P}\underline{\mathbf{v}}_k]_{a_m}} \\ &= -d_m. \end{aligned} \tag{102}$$

□

Corollary 2. *Consider the CTMC defined in Section 1. Since* $\mathbf{Q}^*$ *is upper triangular, its eigenvalues are the diagonal entries. This, together with the fact that* $q_{00} > q_{ii}$ *for any* $i \neq 0$ *gives*

$$-d_m = 2(u_r + zu_c).$$

References

[1] Ohno S. The enormous diversity in genome sizes of fish as a reflection of nature's extensive experiments with gene duplication. Transactions of the American Fisheries Society. 1970;99(1):120–130.

[2] Lynch M, Conery JS. The evolutionary fate and consequences of duplicate genes. Science. 2000;290(5494):1151–1155.

[3] Innan H, Kondrashov F. The evolution of gene duplications: classifying and distinguishing between models. Nature Reviews Genetics. 2010;11(2):97–108.

[4] Liberles DA, Kolesov G, Dittmar K. Understanding gene duplication through biochemistry and population genetics. Evolution After Gene Duplication Hoboken (NJ): Wiley-Blackwell. 2010;.

[5] Force A, Lynch M, Pickett FB, Amores A, Yan Yl, Postlethwait J. Preservation of duplicate genes by complementary, degenerative mutations. Genetics. 1999;151(4):1531–1545.

[6] Lynch M, Force A. The probability of duplicate gene preservation by subfunctionalization. Genetics. 2000;154(1):459–473.

[7] Lynch M, O'Hely M, Walsh B, Force A. The probability of preservation of a newly arisen gene duplicate. Genetics. 2001;159(4):1789–1804.

[8] Hughes T, Liberles DA. The pattern of evolution of smaller-scale gene duplicates in mammalian genomes is more consistent with neo-than subfunctionalisation. Journal of molecular evolution. 2007;65(5):574–588.

[9] Lynch M, Conery JS. The origins of genome complexity. science. 2003;302(5649):1401–1404.

[10] Hughes T, Liberles DA. Whole-genome duplications in the ancestral vertebrate are detectable in the distribution of gene family sizes of tetrapod species. Journal of molecular evolution. 2008;67(4):343–357.

[11] Teufel AI, Zhao J, O'Reilly M, Liu L, Liberles DA. On mechanistic modeling of gene content evolution: birth-death models and mechanisms of gene birth and gene retention. Computation. 2014;2(3):112–130.

[12] Konrad A, Teufel AI, Grahnen JA, Liberles DA. Toward a general model for the evolutionary dynamics of gene duplicates. Genome biology and evolution. 2011;3:1197.

[13] Latouche G, Ramaswami V. Introduction to matrix analytic methods in stochastic modeling. Philadelphia, PA: Society for Industrial and Applied Mathematics; 1999.

[14] Ross SM. Introduction to probability models. 11th ed. Oxford: Academic press; 2014.

[15] Prentice RL, Kalbfleisch JD, Peterson Jr AV, Flournoy N, Farewell V, Breslow N. The analysis of failure times in the presence of competing risks. Biometrics. 1978;p. 541–554.

[16] Bladt M, Neuts MF. Matrix-Exponential Distributions: Calculus and Interpretations via Flows. Stochastic Models. 2003;19(1):113–124.

[17] Bean NG, Fackrell M, Taylor P. Characterization of matrix-exponential distributions. Stochastic Models. 2008;24:339–363.

[18] Kuha J. AIC and BIC comparisons of assumptions and performance. Sociological Methods & Research. 2004;33(2):188–229.

[19] Liberles DA, Teufel AI, Liu L, Stadler T. On the need for mechanistic models in computational genomics and metagenomics. Genome biology and evolution. 2013;5(10):2008–2018.

[20] Hudson D. Interval estimation from the likelihood function. Journal of the Royal Statistical Society Series B (Methodological). 1971;p. 256–262.

[21] Yates A, Akanni W, Amode MR, Barrell D, Billis K, Carvalho-Silva D, et al. Ensembl 2016. Nucleic acids research. 2016;44(D1):D710–D716.

[22] Sefideh FA, Moon MJ, Yun S, Hong SI, Hwang JI, Seong JY. Local duplication of gonadotropin-releasing hormone (GnRH) receptor before two rounds of whole genome duplication and origin of the mammalian GnRH receptor. PloS one. 2014;9(2):e87901.

[23] Uversky VN. Unusual biophysics of intrinsically disordered proteins. Biochimica et Biophysica Acta (BBA)-Proteins and Proteomics. 2013;1834(5):932–951.
