# Supplementary material for: Analysis of a mechanistic Markov model for gene duplicates evolving under subfunctionalization
Source: BMC Evol Biol. 2017 Jan 31;17:38. doi: 10.1186/s12862-016-0848-0 (PMC5282866; doi:10.1186/s12862-016-0848-0)
Supplement: Additional file 2 — Contains the data set analyzed in this work. The data was originally published and analyzed by Hughes and Liblerles [8]. (ZIP 120 kb) [file 12862_2016_848_MOESM2_ESM.zip › Data_HughesLiberles2007/supMat_HughesLiberles2007.pdf]

Supplementary materials to research article: The pattern of evolution of smaller-scale gene duplicates in mammalian genomes is more consistent with neo- than sub-functionalisation

Timothy Hughes and David A. Liberles

TH: Computational Biology Unit, BCCS, University of Bergen, 5020 Bergen, Norway.
Telephone: (+47) 55 58 40 72. Email: tim@bccs.uib.no

DAL: Department of Molecular Biology, University of Wyoming, Laramie, WY 82071, USA.
Telephone: (+1) 307 766 5206. Email: liberles@uwyo.edu.

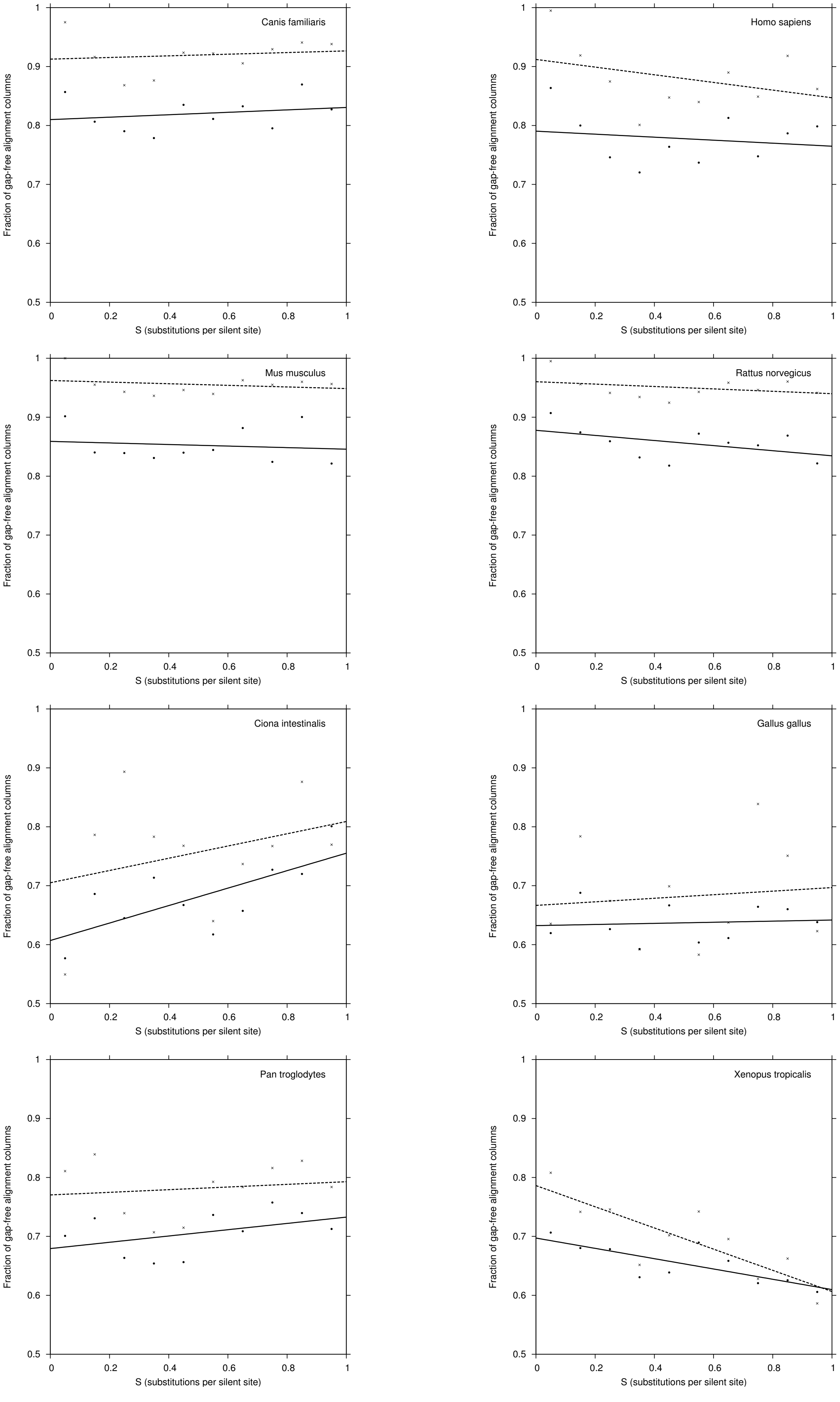

Figure 1: Alignment quality control (fraction of gap-free alignment columns)

Duplicate pairs are placed in groups of size 0.1 S. *Crosses:* group median. *Points:* group mean. *Dotted line:* linear equation fitted to median data. *Full line:* linear equation fitted to mean data.

Species	Duplications per 0.01S	Genes per genome	Duplications per gene per S
C. familiaris	147	18201	0.808
H. sapiens	460	22218	2.070
M. musculus	805	24460	3.291
R. norvegicus	246	21952	1.121

Table 1: Gene duplication rate estimates

Species	Subst. per silent site per BY
C. familiaris	2.94
H. sapiens	2.20
M. musculus	6.07
R. norvegicus	6.07

Table 2: Silent substitution rate estimates

No estimate was available for *C. familiaris*, so the artiodactyl rate was used as this is the nearest lineage for which an estimate was available.

The R code used for fitting the two models to the data and datasets for *C. familiaris* are available online at www.cbu.uib.no/~tim/projects/geneDeathModelling. File paths (to model and dataset files) set in the main R script files will need to be adjusted to the correct values. The file names and a description of their contents are as follows:

supMat/replacementSubstModeling:

bestAltSplices.tab (dataset)

model.repl.low.hughes (model)

outputFunctions.r (functions called from the main R script)

replSubsFitting.r (main R script)

supMat/survivalModeling:

model.survival (model)

outputFunctions.r (functions called from the main R script)

silentSubstCounts_bucketSize_0.01_median.tab (dataset)

survivalFitting.r (main R script)

A description of the columns of the bestAltSplices.tab file (each row contains the data for one duplicate pair):

1 pair ID

2 number of codons in the alignment

3 number of gap free columns in the alignment

4 maximum likelihood with ω estimated

5 maximum likelihood with $\omega = 1$

6 replacement substitutions per replacement site (R) under model where ω estimated

7 silent substitutions per silent site (S) under model where ω estimated

8 replacement sites under model where ω estimated

9 silent sites under model where ω estimated

10 replacement substitutions per replacement site (R) under model where $\omega = 1$

11 silent substitutions per silent site (S) under model where $\omega = 1$

12 replacement sites under model where $\omega = 1$

13 silent sites under model where $\omega = 1$

14 Ensembl ID of first protein in pair

15 Ensembl ID of second protein in pair

A description of the columns of the silentSubstCounts_bucketSize_0.01_median.tab file (each row contains the summary data for one group of duplicate pairs where a groups consist of all duplicate pairs with S within the interval of size 0.01):

1 median S value for the group

2 number of duplicate pairs in the group
